# Fundamental Perspectives on the Electrochemical Water Applications of Metal–Organic Frameworks

**DOI:** 10.1007/s40820-023-01124-3

**Published:** 2023-06-07

**Authors:** Xiang He

**Affiliations:** Department of Mechanical and Civil Engineering, Florida Institute of Technology, Melbourne, FL 32901 USA

**Keywords:** Water remediation, Electrochemistry, Local structures, Pair distribution function, Redox-active MOFs

## Abstract

**Highlights:**

- The recent development and implementation of metal–organic frameworks (MOFs) and MOF-based materials in electrochemical water applications are reviewed.
- The critical factors that affect the performances of MOFs in the electrochemical reactions, sensing, and separations are highlighted.
- Advanced tools, such as pair distribution function analysis, are playing critical roles in unraveling the functioning mechanisms, including local structures and nanoconfined interactions.

**Abstract:**

Metal–organic frameworks (MOFs), a family of highly porous materials possessing huge surface areas and feasible chemical tunability, are emerging as critical functional materials to solve the growing challenges associated with energy–water systems, such as water scarcity issues. In this contribution, the roles of MOFs are highlighted in electrochemical-based water applications (i.e., reactions, sensing, and separations), where MOF-based functional materials exhibit outstanding performances in detecting/removing pollutants, recovering resources, and harvesting energies from different water sources. Compared with the pristine MOFs, the efficiency and/or selectivity can be further enhanced via rational structural modulation of MOFs (e.g., partial metal substitution) or integration of MOFs with other functional materials (e.g., metal clusters and reduced graphene oxide). Several key factors/properties that affect the performances of MOF-based materials are also reviewed, including electronic structures, nanoconfined effects, stability, conductivity, and atomic structures. The advancement in the fundamental understanding of these key factors is expected to shed light on the functioning mechanisms of MOFs (e.g., charge transfer pathways and guest–host interactions), which will subsequently accelerate the integration of precisely designed MOFs into electrochemical architectures to achieve highly effective water remediation with optimized selectivity and long-term stability.
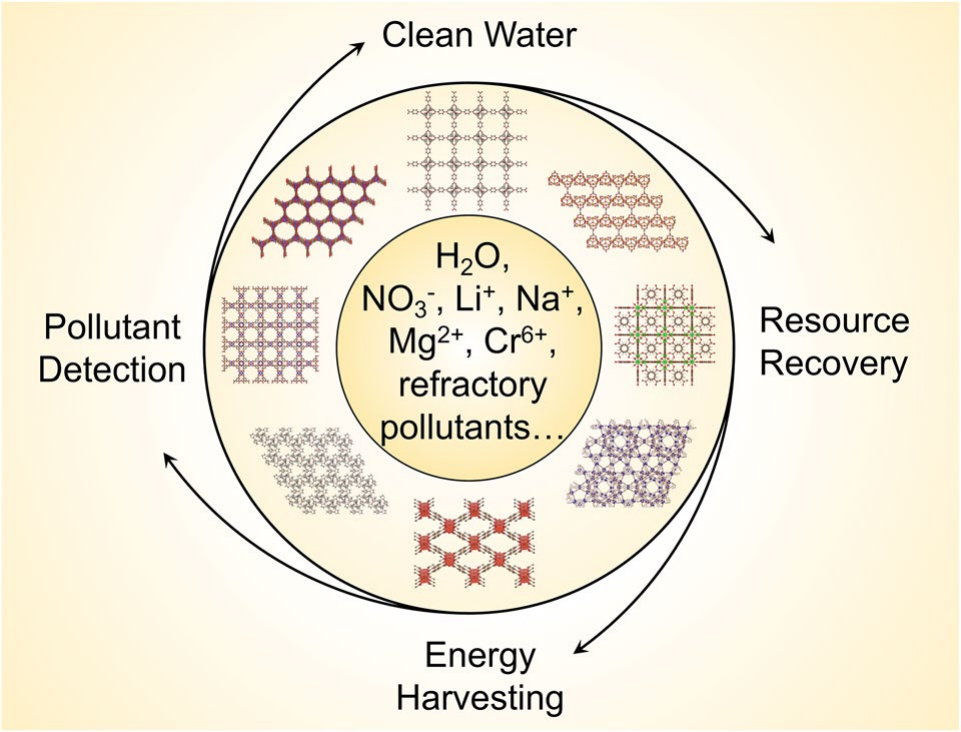

## Introduction

In recent decades, water scarcity has been becoming server due to the rapid increase in water consumption and local populations, which has expanded the percentage of the global population suffering from water scarcity from 14% in the 1900s (*i.e.*, ~ 200 million) to 58% by the 2000s (*i.e.*, 3.8 billion) [1]. Effective water treatment is among the possible avenues to address water scarcity issues, which can provide additional resources to balance water demand and availability and further contribute to the circular economy [2]. The quest for efficient water treatment has led to the discovery, development, and implementation of a variety of advanced functional materials, including but not limited to graphene [3], graphitic carbon nitride [4], MXene [5], and metal–organic frameworks (MOFs) [6].

**Fig. 1:**
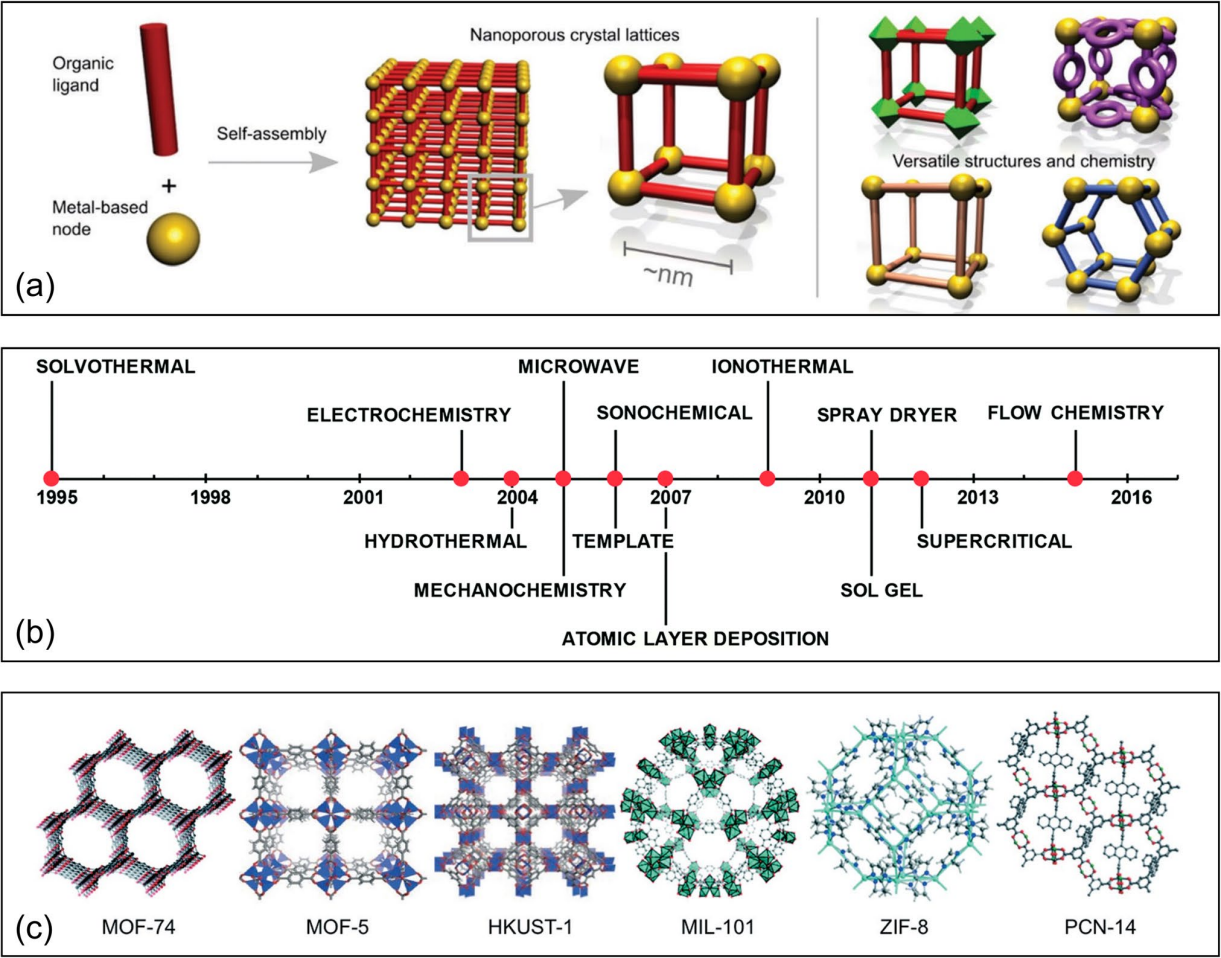
**a** Schematic illustration of MOF structures. Reproduced from Ref. [7] with permission from the Royal Society of Chemistry. **b** Methods to synthesize MOFs on a timeline. Reproduced from Ref. [8] with permission from the Royal Society of Chemistry. **c** Representative MOF structures. Reproduced from Ref. [14] with permission from the Royal Society of Chemistry

Among these functional materials, MOFs are standing out as emerging and multifunctional materials, which synergistically integrate functionalities and benefits of both discrete molecules and extended solids. MOFs are constructed from metal-based nodes and organic linkers, forming highly ordered porous structures, where the vast metal/linker species permit the design of versatile architectures (Fig. 1a) [7]. Over the past three decades, a wide range of approaches has been developed for the synthesis of MOFs (Fig. 1b) [8], including but not limited to the solvothermal method, hydrothermal method, and spray drying. For example, the solvothermal method produces MOF crystals by heating the mixture of metal ions, organic linkers, and solvents in sealed vessels typically at temperatures higher than the boiling points of the solvents [9]. The formation of MOF crystals can be time-consuming and can be facilitated using different energy sources, such as microwaves [10] and ultrasonic irradiation [11]. As a newly developed strategy for MOF synthesis, the spray drying processes leverage microdroplets as mini reactors for MOF growth [12, 13], which addresses the slow heat and mass transfer issues associated with MOF synthesis in bulk solutions, allowing for the rapid and facile design of multifunctional MOF-based materials. Several representative MOF structures are shown in Fig. 1c [14], such as HKUST-1 (HKUST: Hong Kong University of Science and Technology), MIL-101 (MIL: Matériaux de l’Institut Lavoisier), ZIF-8 (ZIF: zeolitic imidazolate frameworks), and PCN-14 (PCN: porous coordination network). Compared with conventional porous functional materials, MOFs possess many remarkable properties, including but not limited to exceptionally large surface areas and molecular-level tunability, which has led to the initial and wide success of MOFs and MOF-derived materials in gas adsorption and separation [15–17], biomedical applications [18, 19], heterogeneous catalysis [20–25], energy storage [26], sensing [27], and more. These exceptional properties of MOFs have also been drawing increasing attention to achieve highly efficient water remediation [28–30], especially those driven by electrochemistry [31, 32], which offers great efficiency, versatility, and compatibility.

The implementation of MOFs in electrochemical water applications brings many technical advantages, such as easy products/materials separation, highly concentrated and well-dispersed active sites, and tunable functionalities, which have resulted in outstanding performances in electrochemical-based reactions, sensing, and separations during water treatment. For example, the redox-active metal ions or linkers in MOFs endow remarkable catalytic activity; meanwhile, the excellent porosities and huge surface areas of MOFs allow for selective capture of target molecules, which synergistically offer amplified signals and considerable sensitivities when MOFs are used for electrochemical sensing [33]. Despite the promising performances, applying MOFs for electrochemical water applications is still at an early stage, and further advances in fundamental understanding are in urgent demand to address challenges related to MOFs’ stability, conductivity, and detailed functioning mechanisms, which require in-depth insights into nanoconfined interactions, atomic structures, and electronic structures.

**Scheme 1:**
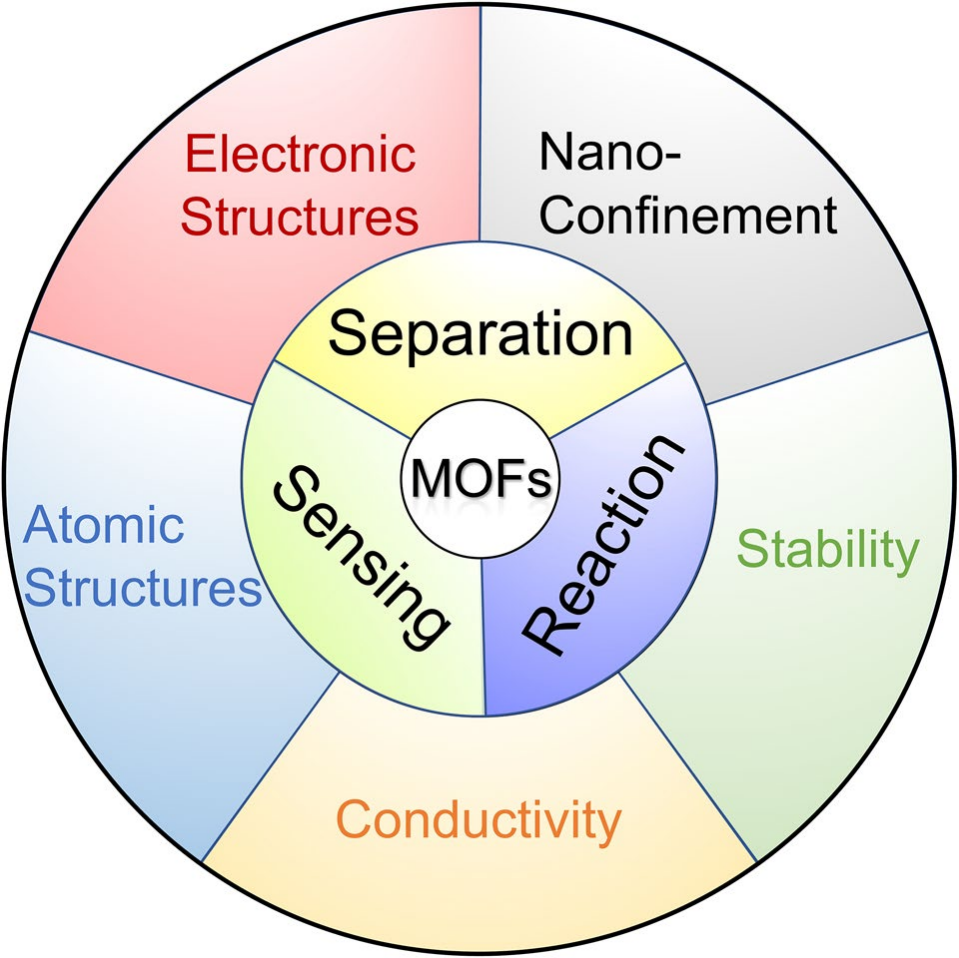
Schematic overview

Currently, many comprehensive reviews have been contributed by the research community regarding a variety of MOFs-related topics, such as the synthesis [8, 34–36], post-synthetic modification [37], and multifunctional composites [38]. However, fewer have focused on the advancement of MOFs in electrochemical water applications with an extensive survey of the limiting factors and properties. Herein, the goal of this contribution is to provide a timely and broad review (Scheme 1) of not only the ongoing developments of MOFs for energy-efficient electrochemical water applications (Sect. 2), including reaction (Sect. 2.1), sensing (Sect. 2.2), and separation (Sect. 2.3), but also the fundamental advancements, challenges, and opportunities in electronic structures (Sect. 3.1), nanoconfinement (Sect. 3.2), electrochemical stability of MOFs (Sect. 3.3), conductivity in MOF systems (Sect. 3.4), and atomic structures of MOFs (Sect. 3.5).

## MOFs in Electrochemical Water Applications

MOFs are being increasingly implemented in electrochemical systems for enhanced water applications through either Faradaic or non-Faradaic processes [32]. Efforts have been directed toward improved performances in sensing/removing pollutants and recovering resources with primary considerations on the MOF’s activity, selectivity, and stability. Some representative demonstrations of using MOFs for electrochemical water applications are listed in Table 1. The following sections will highlight the implementations and roles of MOFs in the reaction, sensing, and separation processes during electrochemical water applications.

**Table 1:** A list of representative MOF-based functional materials for electrochemical water applications

Materials	Electrochemical functions	Performance	References
CuHHTP with Cu clusters (HHTP: 2,3,6,7,10,11-hexahydroxytriphenylene)	Nitrate reduction	Nitrate conversion rate: 85.81%; ammonia selectivity: 96.84%	[42]
Cu@Th-BPYDC (BPYDC: 2,2′-bipyridine-5,5′-dicarboxylic acid)	Nitrate reduction	Faradaic efficiency: 92.5%; ammonia production yield: 225.3 μmol h^−1^ cm^−2^	[48]
[(CH_3_)_2_NH_2_][In(TTFOC)] (In8) (TTFOC: tetrathiafulvaleneoctacarboxylate)	Nitrate reduction	Faradaic efficiency: 90.1%; ammonia production yield: 278.8 μg h^−1^ cm^−2^ ; ammonia selectivity: 99.3%	[49]
S-doped MIL-53 (MIL: Matériaux de l’Institut Lavoisier)	Electro-Fenton catalysis	Turnover frequency: 0.48 L g^−1^ min^−1^ ; removing 95.8% sulfamethazine	[52]
MIL-88A with reduced graphene oxide and carbon felt	Electro-Fenton catalysis	Pollutant degradation within 120 min: 91.4%	[56]
MOF-525	Nitrite sensing	Detection limit: 2.1 μM; linear range: 20–800 μM; sensitivity: 95 μA/mM-cm^2^	[64]
Ag-SO3-NU-902 (NU: Northwestern University)	Nitrite sensing	Detection limit: 9.1 μM; linear range: up to 2 mM	[47]
UiO-66 (UiO: Universitetet i Oslo)	2,4,6-Trichlorophenol sensing	Detection limit: 1.29 μg L^−1^	[72]
Hierarchical Cu-BTC (BTC: benzene-1,3,5-tricarboxylic acid)	Glyphosate sensing	Detection limit: 1.4 × 10^–13^ mol L^−1^ ; linear range: 10^–12^ to 10^–9^ mol L^−1^ and 10^–9^ to 10^–5^ mol L^−1^	[71]
Cr-MIL-101	Perfluorooctanesulfonate sensing	Detection limit: 0.5 ng L^−1^	[67]
Mn(TPA)-SWCNTs composite (TPA: terephthalic acid; SWCNTs: single-walled carbon nanotubes)	Pb(II) sensing	Detection limit: 38 nM; linear range: 0.1–14 μM	[69]
Bi(III)/MIL-101(Cr)	Pb(II) and Cd (II) sensing	Detection limit: 60 ng L^−1^ (Cd (II)) and 70 ng L^−1^ (Pb(II)); linear range: 0.1–30 and 30–90 μg L^−1^	[65]
Cu-MOF-74	Cd (II) removal	Adsorption capacity: > 0.9 mmol g^−1^	[77]
Cl-IIP@UiO-66 (Cl-IIP: chlorine ion-imprinted polymer)	Ion separation	High separation factors: Cl^−^/Br^−^: 9.02; Cl^−^/F^−^: 10.29, Cl^−^/SO_4_^2−^: 13.41; Cl^−^/PO_4_^3−^: 18.50	[79]
Polypyrrole/HKUST-1 (HKUST: Hong Kong University of Science and Technology)	Lithium extraction	Adsorption capacity: 37.55 mg g^−1^ ; adsorption equilibrium time: < 25 min	[80]
Polystyrene sulfonate @ HKUST-1	Lithium extraction	Lithium conductivity: 5.53 × 10^–4^ S cm^−1^ (25 °C) and 1.89 × 10^–3^ S cm^−1^ (70 °C); ideal selectivities: Li^+^/Na^+^: 78, Li^+^/K^+^: 99, Li^+^/Mg^2+^: 10,296	[81]
UiO-66-NH_2_ @ ANM (ANM: alumina nanochannel membrane)	Osmotic energy harvesting	Power density: up to 26.8 W m^−2^	[75]
Zn-TCPP/AAO (TCPP: 5,10,15,20-Tetra(4-carboxyphenyl)porphyrin; AAO: anodic aluminum oxide)	Osmotic energy harvesting	Power density: 6.26 W m^−2^ (dark) and 7.74 W m^−2^ (light)	[86]

### Reaction: Nitrate Reduction and Pollutant Degradation

The electrochemical reactions can serve as an effective and sustainable method to detoxify water contaminants or convert them into useful resources [39]. In principle, MOFs can be used as matrix frameworks to host electroactive species or leverage the redox-active building units as catalytic centers to achieve promising performances in redox-based electrochemical water remediations, such as electrocatalytic nitrate reduction [40] and degradation of organic pollutants [41]. Several representative studies are presented as follows.

The engineering potentials of MOFs have been explored in the electrocatalytic nitrate (NO_3_^−^) reduction process, which offers not only energy-efficient production of ammonia (NH_3_) but also effective removal of nitrate contamination from water [42]. In particular, the nitrate concentration in wastewater can reach 2 M [40], which can result in serious risks to ecosystems. As a result, the World Health Organization requires nitrate concentration in drinking water to be below 10 mgN L^−1^ [43]. Nitrate can be removed from the aqueous environments by reducing nitrate to nitrogen (Eq. 1, ${E}^{o}=1.17V vs. SHE$) or ammonia (Eq. 2, ${E}^{o}=-0.12V vs. SHE$) [44].

1 $$2{{\mathrm{NO}}_{3}}^{-}+12{\mathrm{H}}^{+}+10{\mathrm{e}}^{-}\to {\mathrm{N}}_{2}+6{\mathrm{H}}_{2}\mathrm{O}$$

2 $${{\mathrm{NO}}_{3}}^{-}+9{\mathrm{H}}^{+}+8{\mathrm{e}}^{-}\to {\mathrm{NH}}_{3}+3{\mathrm{H}}_{2}\mathrm{O}$$

**Fig. 2:**
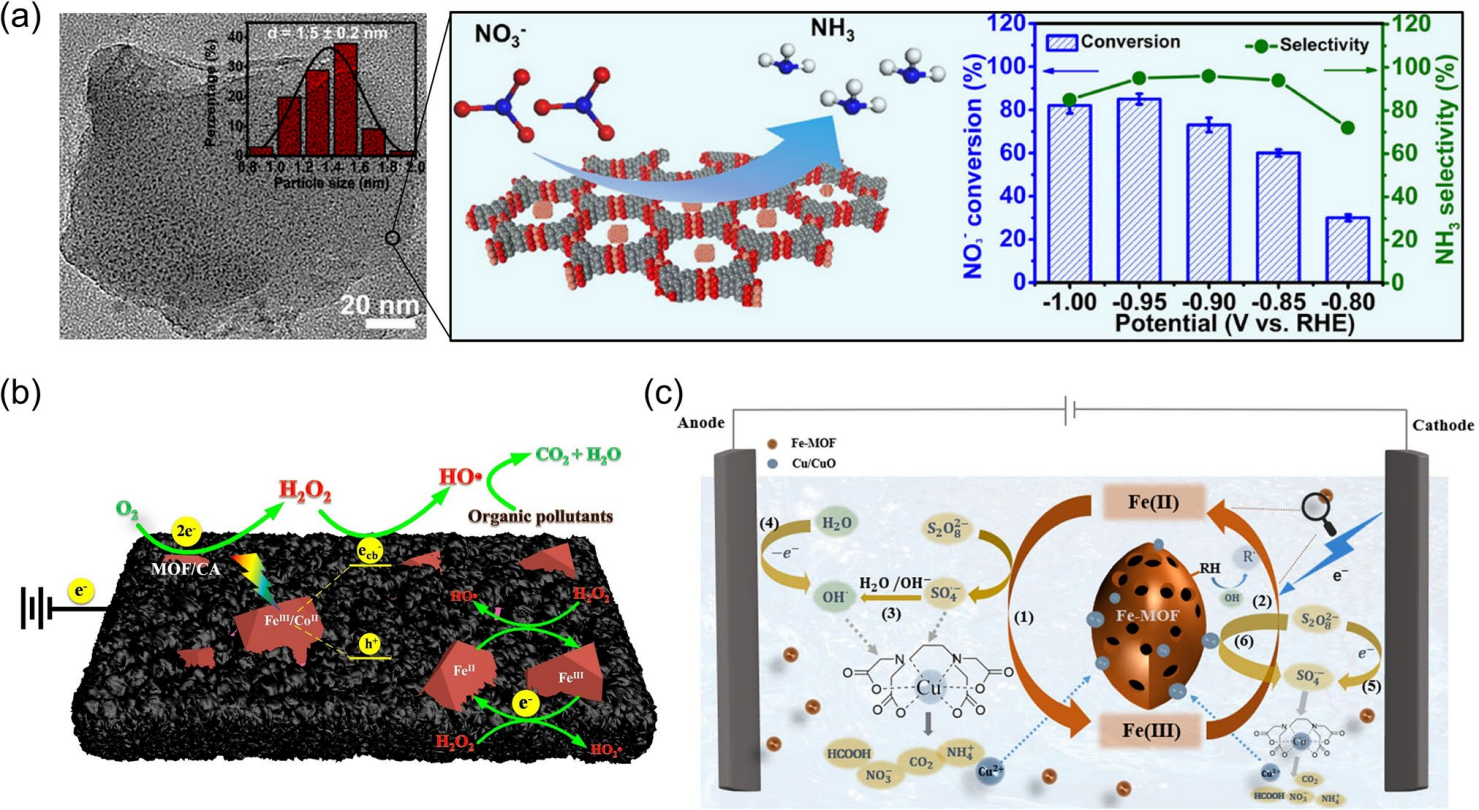
**a** Composites of CuHHTP (*i.e.*, a conductive MOF) and confined Cu clusters (from left to right: TEM image, composite scheme, and electrocatalytic performance to convert NO_3_^−^ to NH_3_. Adapted with permission from Ref. [42]. Copyright 2022 American Chemical Society. **b** A composite cathode of carbon aerogel and bimetallic MOFs is used for the photo-electro-Fenton degradation of organic pollutants. Reprinted from Ref. [50], Copyright (2016), with permission from Elsevier. **c** An electrochemical system utilizing a Fe-based MOF and persulfate for the degradation of Cu-EDTA. Reprinted from Ref. [57], Copyright (2021), with permission from Elsevier

Unlike conventional biological denitrification [45], which primarily reduces nitrate to nitrogen, the MOF-based electrochemical processes prefer to form ammonia, which is a value-added product that can serve as an essential agricultural ingredient and an energy storage carrier [46], offering additional benefits beyond the water remediation. In a recent work by Zhu et al*.* [42], a conductive MOF constructed from Cu and HHTP (HHTP = 2,3,6,7,10,11-hexahydroxytriphenylene) was used to host ultrafine Cu clusters (~ 1.5 nm), and this composite material demonstrated a high nitrate conversion rate (*i.e.*, 85.81%) with an ammonia selectivity of 96.84% (Fig. 2a), which stem from the unique properties of Cu clusters, including the “accept-donate” charge transfer mechanism and the high d-band center. Through a tandem procedure of the post-synthetic modification and ion exchange, Wang et al*.* [47] successfully immobilized Ag(I) ions within the framework of NU-902 (a Zr-based MOF, NU: Northwestern University) using the sulfonate-based ligands, along with ~ 3 nm silver nanoparticles. In this Ag-SO3-NU-902 composite system, both the confined silver nanoparticles and the porphyrinic linkers are effective in the electro-oxidation of nitrite, making this composite structure a potential nitrite sensor with a limit of detection of 9.1 μM and promising selectivity. In addition to the metal clusters and nanoparticles, molecules with single metal sites can also be incorporated into the MOF matrix for efficient nitrate reduction. Taking the thorium-based MOF (Th-MOF) as an example, single Cu^II^ sites can be affixed within Th-MOF through post-synthetic metalation, offering open metal sites for the enhanced electroreduction of nitrate to ammonia with remarkable Faradaic efficiency of 92.5% [48]. Other than the catalytic sites of the guest species, MOFs have intrinsically well-dispersed metal nodes within the framework, which could potentially be used as catalytic centers for nitrate electroreduction. For example, Lv et al. [49] designed a two-dimensional In-based MOF catalyst, where ligands can be partially dissociated in a reversible process, which can generate unsaturated In^II^ reactive sites, functioning like highly condensed, single-atom catalytic systems for the eight-electron conversion of nitrate to ammonia.

Due to their high effectiveness and environmental compatibility, electro-Fenton processes are among the most powerful advanced oxidation processes (AOPs) for unselective pollutant degradation. During the electro-Fenton processes, Fe^2+^ ions activate hydroperoxides (H_2_O_2_) into hydroxyl radicals (${OH}^{\bullet }$) (Eq. 3) [32], which are highly oxidative and capable of mineralizing organic pollutants on a non-selective basis. After that, Fe^2+^ ions will be regenerated through either the reduction of Fe^3+^ at the cathode (Eq. 4) or the reaction between Fe^3+^ and H_2_O_2_ (Eq. 5), which produces protons and hydroperoxyl radicals (${{HO}_{2}}^{\bullet }$) [32, 50]. With a sufficient supply of H_2_O_2_, these reactions can continuously provide reactive oxygen species (ROS) for water remediation.

3 $${\mathrm{Fe}}^{2+}+{\mathrm{H}}_{2}{\mathrm{O}}_{2}\to {\mathrm{Fe}}^{3+}+{\mathrm{OH}}^{-}+ {\mathrm{OH}}^{\bullet }$$

4 $${\mathrm{Fe}}^{3+}+{\mathrm{e}}^{-}\to {\mathrm{Fe}}^{2+}$$

5 $${\mathrm{Fe}}^{3+}+{\mathrm{H}}_{2}{\mathrm{O}}_{2}\to {\mathrm{Fe}}^{2+}+{\mathrm{H}}^{+}+ {{\mathrm{HO}}_{2}}^{\bullet }$$

6 $${\mathrm{O}}_{2}+ {2\mathrm{e}}^{-}+ {2\mathrm{H}}^{+}\to {\mathrm{H}}_{2}{\mathrm{O}}_{2}$$

One additional advantage is that the electro-Fenton process can be incorporated with the in situ generation of H_2_O_2_ through the oxygen reduction reactions on the cathode (Eq. 6), which greatly reduces the cost and safety concerns regarding storage and transportation [32]. Recent attention has been directed to MOFs-based electro-Fenton reactions [51]. For example, research has reported on the MOFs design with tandem electrochemical functions of H_2_O_2_ generation followed by H_2_O_2_ activation to •OH [32]. Zhao et al. [50] recently designed a composite cathode using a bimetallic MOF(Fe/Co) and carbon aerogel, which was incorporated into a photo-electro-Fenton process, where the carbon drove the in situ H_2_O_2_ generation via the oxygen reduction reactions, while the bimetallic MOF(Fe/Co) completed the catalytic conversion of H_2_O_2_ to hydroxyl radicals (Fig. 2b). With the optimal Fe/Co ratio of 2:1, this composite electrode exhibited excellent performances in removing dimethyl phthalate and rhodamine B over a wide pH range. Du et al. [52] demonstrated that the Fe-based MIL-53 can be sulfurized to produce S-modified MIL-53(Fe), which possesses an enlarged surface area and increased content of Fe^2+^. The resultant S-MIL-53(Fe) showed a high efficiency as a heterogeneous electro-Fenton catalyst with a turnover frequency of 0.48 L g^−1 ^min^−1^, a 6.8-fold enhancement when compared with the commercial FeS_2_, which led to the effective generation of ${OH}^{\bullet }$ from H_2_O_2_ and removal of 95.8% of the sulfamethazine at the neutral pH. A composite catalyst of MOFs(Ce)/Fe_3_O_4_@C was designed by Su et al. [53], which can be optimized to create large content of Fe^2+^, Ce^3+^, and oxygen vacancies, synergistically promoting the formation of various reactive oxygen species (*e.g.*, superoxide anion (O_2_^•−^) and hydroxyl radical (${\mathrm{OH}}^{\bullet }$)) during the heterogeneous electro-Fenton reactions that offer a high efficiency toward the removal of sulfamethazine with minimal energy consumption. Wang et al. [54] constructed a composite membrane using a Fe/Co-based bimetallic MOF and polyacrylonitrile-derived carbon nanofibers. The resultant composite membrane can be used as a cathode and integrated into the photo-electro-Fenton system, which can continuously generate the hydroxyl radicals following the in situ H_2_O_2_ formation via the oxygen reduction reaction. As a result, the system was capable of degrading 99% of perfluorooctanoic acid (PFOA) with a mineralization efficiency of 91% after 120 min, where the Co/Fe valence state change played a vital role. Similarly, biomass-based carbon fibers can also serve as the support of functional MOFs for the rational design of electro-Fenton reactions [55]. For example, the carbonization of the cotton fiber membrane can produce carbon fiber membrane, which can serve as the electrode support for the introduction of bimetallic functional MOF particles via the solvothermal method [55]. The as-prepared hybrid electrode exhibited excellent efficiency in generating hydroxyl radicals and degrading the organic pollutant of tetrabromobisphenol A (TBBPA) (*i.e.*, 80% removal efficiency within 4 h). In addition, Xie et al. [56] developed a ternary cathode for the electro-Fenton reactions, which was fabricated using carbon felt, reduced graphene oxide, and a Fe-based MIL-88A. This ternary cathode system showed an enhanced charge transfer rate as well as effective H_2_O_2_ formation and activation to produce various reactive oxygen species for the degradation of pollutants, where the reversible Fe^2+^/Fe^3+^ redox reactions made positive contributions. In addition to H_2_O_2_, the Fe-based MOFs can also be coupled with other chemicals to generate ROS, such as persulfate [57]. For example, Zhang et al. [57] applied the microwave method to synthesize a ferreous MOF, which can be combined with persulfate to produce ROS under the electrochemical processes (Fig. 2c). With the optimal parameters (*e.g.*, pH and persulfate dosage), this reactive system was able to completely remove a refractory pollutant (*i.e.*, copper–ethylenediaminetetraacetic acid, Cu-EDTA) after 100 min of reaction. In addition to the particle form, MOFs-based functional materials can also be integrated into other platforms. In a recent study, Zhou et al*.* [58] fabricated an anti-fouling membrane by incorporating conductive graphite particles and defective UiO-66 (UiO: Universitetet i Oslo) into the polyvinylidene fluoride (PVDF) matrix. When used as an anode with the 0.01 mA cm^−2^ current density, the as-prepared membrane proved promising self-cleaning and anti-fouling capabilities under both continuous and intermittent conditions. In particular, the excellent electrochemical activity of the membrane allowed the effective generation of free radicals (*e.g.*, O_2_^•−^ and ${\mathrm{OH}}^{\bullet }$), which offered superior performance in removing antibiotics and bacteria (*i.e.*, *E. coli* and *S. aureus*).

Overall, the aforementioned studies have demonstrated the effectiveness of MOFs in electrochemical water remediation involving redox reactions (*e.g.*, nitrate reduction and electro-Fenton reactions), which can be achieved by (1) incorporating redox-active species (*e.g.*, Cu clusters, Ag nanoparticles, and single Cu^II^ sites) into the MOF matrices and (2) building MOFs with redox-active linkers (*e.g.*, porphyrinic linkers) and metal nodes (*e.g.*, open In^II^ sites). In particular, the iron-based MOFs can also be integrated with H_2_O_2_ or persulfate, which utilizes the reversible Fe^2+^/Fe^3+^ redox reactions to continuously generate the reactive radicals, such as ${\mathrm{OH}}^{\bullet }$ and O_2_^•−^, holding remarkable potentials for the unselective degradation of the refractory pollutants in water, including but not limited to PFOA, TBBPA, and Cu-EDTA. To promote the efficiency of MOFs in these redox reactions, structural modifications can be made to the MOFs, such as creating atomic vacancies, generating unsaturated metal sites, or incorporating secondary metal sites, as demonstrated in the aforementioned studies. In another route, the reactivity and performance of the MOFs-based electroactive materials can also be further enhanced by carbon-related functional materials (*e.g.*, carbon fibers and reduced graphene oxide). It is also expected that the distinct features of MOFs will continue to play a crucial role in the electrochemical water remediation, including tunable functionalities of MOFs, the existence of bimetallic nodes, reversible redox activities, and nanoconfined effects posed on the guest metal clusters, which will make MOFs-based functional materials promising solutions to remediate water using electrochemical reactions in an efficient, sustainable, and environmentally compatible route. Despite the promising outcomes regarding MOF-based electrochemical water remediation, fundamental studies are still very rare in identifying the active sites, dynamic structural changes, charge transfer kinetics/pathways, reaction mechanisms, etc. Therefore, additional work is mandated to establish the comprehensive relationship among MOFs’ structures, properties, and redox functionalities.

### Sensing Toxic Substances in Water

The outstanding properties also make MOFs highly promising platforms for electrochemical sensing [59, 60]. In particular, MOFs’ huge porosities and surface areas can provide large sensing interfaces to the efficiently concentrated analytes, which results in boosted electrochemical signal response and detection sensitivity. In the meantime, the structural diversity of MOFs, such as a variety of cavities and channels, offers tunable molecular sieve effects for improved detection selectivity, which can be further enhanced via modification of the active sites (*i.e.*, metals and ligands). Given the aforementioned advantages of MOFs for electrochemical sensing, considerable interest has been raised in using MOF-based electrodes to detect toxic substances in water [61], including but not limited to heavy metal ions (*e.g.*, AS^3+^ and Pb^2+^), nitrite, pesticides, and antibiotics [62, 63].

**Fig. 3:**
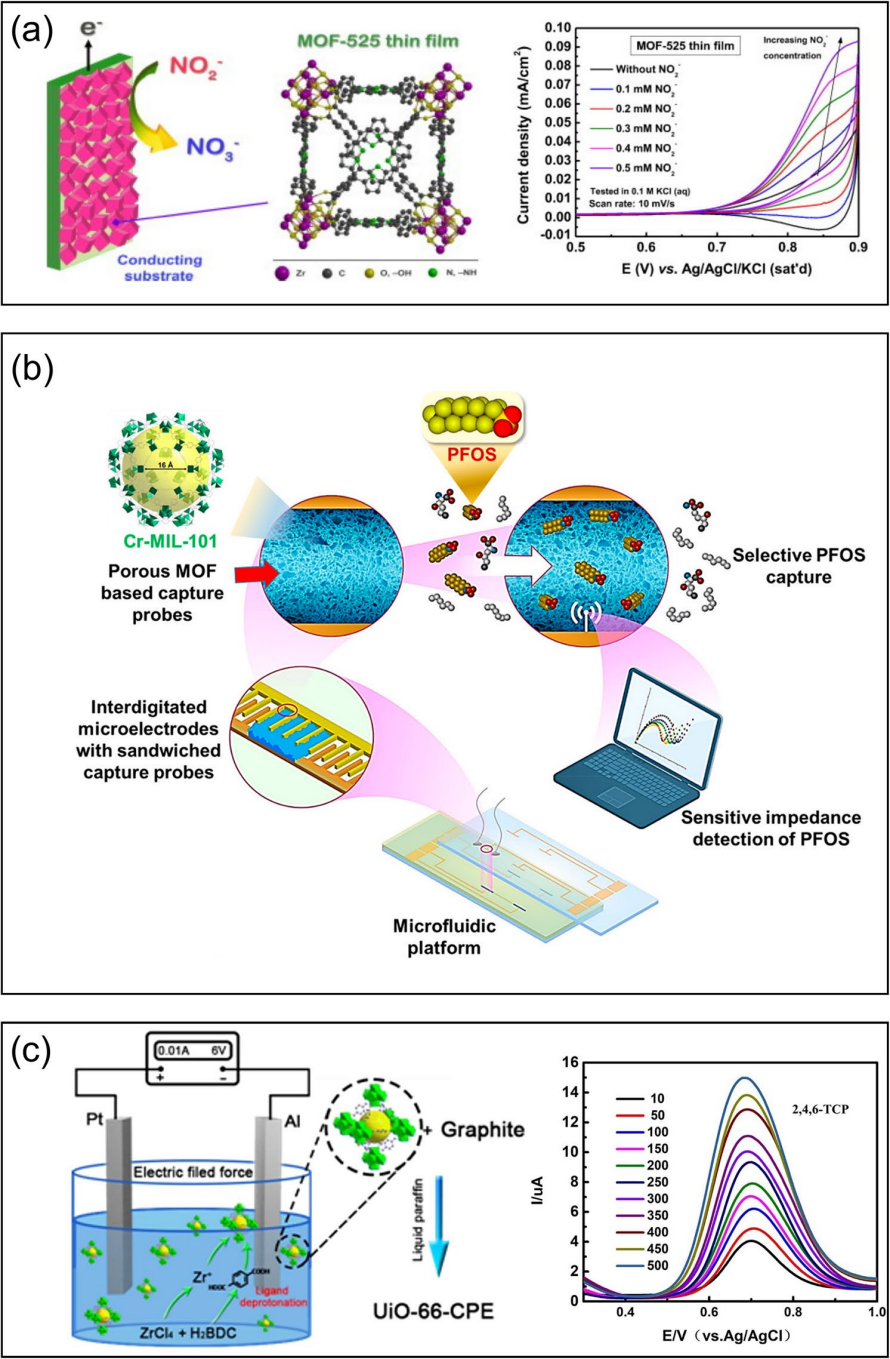
**a** Electrochemical detection of nitrite using the Zr/porphyrin-based MOF (*i.e*., MOF-525). Reprinted from Ref. [64], Copyright (2015), with permission from Elsevier. **b** Scheme of a microfluidic platform using Cr-MIL-101 for the perfluorooctanesulfonate detection. Reprinted with permission from Ref. [67]. Copyright 2020 American Chemical Society. **c** Electrochemical manufacturing of the UiO-66-based electrode for the 2,4,6-trichlorophenol detection using differential pulse voltammetry. Reprinted from Ref. [72], Copyright (2019), with permission from Elsevier

For instance, Kung et al*.* [64] recently developed an amperometric nitrite (NO_2_^−^) sensor using uniform thin films of the zirconium–porphyrin-based MOF-525 (Fig. 3a). The porphyrin-based linkers of MOF-525 can be electrochemically oxidized to the cation radical state, which can serve as redox-active centers for nitrite oxidation. Compared with the bare conductive substrate, the presence of the MOF-525 improved the electrochemical oxidation of NO_2_^−^ by more than two orders of magnitude. With the amperometric technique, this MOF-525-based thin film showed a promising capability to detect NO_2_^−^ in water over a wide concentration range with a detection limit and sensitivity of 2.1 μM and 95 *μ*A/mM-cm^2^, respectively. In a recent study, Shi et al*.* [65] employed electrochemical reduction to generate metallic bismuth inside the matrix of MIL-101(Cr). With the carbon cloth electrode (CCE) as the conductive support, the resultant Bi/MIL-101(Cr)/CCE exhibited the reproducible and promising capability of sensing trace Cd(II) and Pb(II) in water with the respective detection limits of 0.06 and 0.07 μg L^−1^, way below the safety requirements of the World Health Organization. This integrated Bi/MIL-101(Cr)/CCE outperformed the individual counterparts, which was mainly due to the combined effects of the excellent porosities of the MIL-101(Cr) matrix, activities of Bi and MIL-100(Cr) as well as the selective metal–ligand interactions. Singh et al*.* [66] recently developed a simple, precise, and sensitive platform to detect trace Hg^2+^ in tap and fish water using cubic Cu-based MOF, which allowed for favorable adsorption and preconcentration of Hg^2+^ due to the porous architecture and affinity of Hg^2+^ to the functional groups (*e.g.*, N–H and O–H), leading to a low detection limit of 0.0633 nM. Cheng et al. [67] designed an analytical platform for the in situ detection of perfluorooctanesulfonate (PFOS) by embedding Cr-MIL-101 into a microfluidic channel (Fig. 3b). As demonstrated through microscopic and spectroscopic analysis (*e.g.*, transmission electron microscopy (TEM) and X-ray photoelectron spectroscopy (XPS)), the Cr centers in Cr-MIL-101 exhibited affinity toward both sulfonate and fluorine groups; meanwhile, the design of the non-planar interdigitated microelectrode secured the electric field penetration across the non-conductive MOF, which synergistically improved the sensitivity with an unprecedented PFOS sensing limit of 0.5 ng L^−1^.

The sensing performance of MOFs can be improved further after addressing their intrinsic property restrictions (*e.g.*, limited conductivity and mass transfer rate) [59]. Taking conductivity as an example, strategies have been developed to promote the charge transfer within MOF-based electrodes, such as combining MOFs with carbon-based materials [68], including but not limited to single-walled carbon nanotubes (SWCNTs) [69] and graphene oxide (GO) [70]. For instance, Cai et al. [69] designed a composite functional material using SWCNTs and manganese-terephthalic acid MOF (Mn(TPA), which synergistically integrated the excellent conductivity of SWCNTs with Mn(TPA)’s ability for the effective and selective adsorption of Pb^2+^. As a result, the SWCNTs/Mn(TPA) exhibited remarkable Pb^2+^ sensing performance (*e.g.*, the limit of detection: 38 nM and linear response over 0.1–14 $\mu$M) and showed promising potential for industrial wastewater applications. Similar to SWCNTs, GO can also be employed to boost the conductivity of MOFs for electrochemical sensing as demonstrated by Ambaye et al*.* [70], where a copper-based MOF (Cu-MOF) was assembled with the nitrogen-doped GO (NGO) via the solvothermal approach. The as-prepared Cu-MOF/NGO composite demonstrated effective analytical performance for the electrochemical sensing of 4-nitrophenol (4-NP, a phenolic derivative and a hazardous contaminant), which was primarily attributed to the enhanced charge transfer kinetics. In addition to carbon-based materials, other conductive materials (*e.g.*, metal nanoparticles and metal oxides) can also be loaded into the MOF matrices to enhance the conductivity [63]. As critical factors for the sensitivity of electrochemical sensing, the accessible surface area as well as the mass and electron transfer can be improved via hierarchical structural design [36, 59]. For example, Cao et al*.* [71] applied the hierarchically porous MOF for the electrochemical sensing of glyphosate, which is a widely used herbicide that is difficult to detect. Benefiting from the hierarchical porosity with both mesoporous and microporous characteristics, the sensor exhibited enhanced detection performance over a wide range, such as low detection limit and good selectivity against other interferences. Moreover, defect engineering is also an emerging strategy to enhance the selectivity and the anti-interference ability of electrochemical sensors, where local heterogeneities are introduced to tune the redox potentials [59]. For instance, Zhang et al. [72] used electrochemical cathode methods to fabricate defects on the Zr clusters in UiO-66, which created unsaturated metal coordination sites that can promote the electrocatalytic activity. As a result, this UiO-66-based electrode demonstrated promising performance in sensing 2,4,6-trichlorophenol (Fig. 3c) with a detection limit of 1.29 $\mu$g L^−1^ (*i.e.*, below the national standard of 10 $\mu$g L^−1^) and a strong anti-interference ability against common ions, phenol, and 2,4-dichlorophenol.

The aforementioned studies have provided clear demonstrations that the huge porosity, structural diversity, and chemical tunability of MOFs can create considerable opportunities for reliable electrochemical sensing of water contaminants, such as heavy metal ions (*e.g.*, Pb^2+^, Cd^2+^, and Hg^2+^) and organic pollutants (*e.g.*, PFOS, antibiotics, and pesticides). In these processes, MOFs can serve in two different roles: (1) as porous matrices that leverage the high surface areas and unique guest–host interactions to concentrate trace contaminants in a selective manner and (2) as redox-active platforms for sensitive detection. The performance of MOFs as electrochemical sensors, such as sensitivity, can be hindered by MOFs’ poor conductivity, which was partially addressed in prior studies through the incorporations of carbon-based materials (*e.g.*, SWCNTs and NGO) or metal nanoparticles. Other than combining MOFs with conductive materials, MOFs can as be integrated with the non-planar interdigitated microelectrode to enhance the penetration of the electric field through the non-conductive MOFs. Furthermore, the current progress on designing electronically conductive MOFs is also expected to contribute to improved sensing performance, which will be discussed in Sect. 3.4. Another important factor for MOF-based electrochemical sensing of water contaminants is the limited mass transport, and the hierarchical structural design with combined mesoporous/microporous seems to be a promising solution. Overall, the MOF-based platforms for the electrochemical sensing of water contaminants have been showing superior performance compared with the conventional functional materials, but additional innovations are still needed to unlock the full potential of MOFs for electrochemical sensing of pollutants in water, where the comprehensive and interactive evaluation of a series of analytical parameters are required, such as sensitivity, selectivity, linear range, as well as anti-interference capability [61, 62].

### Separation for Water Remediation and Resource/Energy Recovery

MOFs have tunable, accessible, and well-defined pore apertures and channels typically with narrow size distributions, offering structural advantages for precise separation applications [73, 74], such as gas separations and liquid-phase separations. In particular, the channel dimensions (*e.g.*, window size and cavity size) of many MOF species are close to the sizes of hydrated ions, making these MOFs promising candidate materials as ionic filters for desalination or osmotic energy harvesting [75]. Overall, the selective ion transport through the angstrom-scale MOFs channels can be accomplished via several major mechanisms, including the size exclusion effect and electrostatic interactions [74, 76]. Overall, there are two approaches to using MOFs for separations under the electric field: (1) design MOFs-based electrodes and (2) develop MOFs-based membranes that can be used as ionic filters under electrodialysis. Some of the recent work on MOFs for electrochemical separation is highlighted below.

**Fig. 4:**
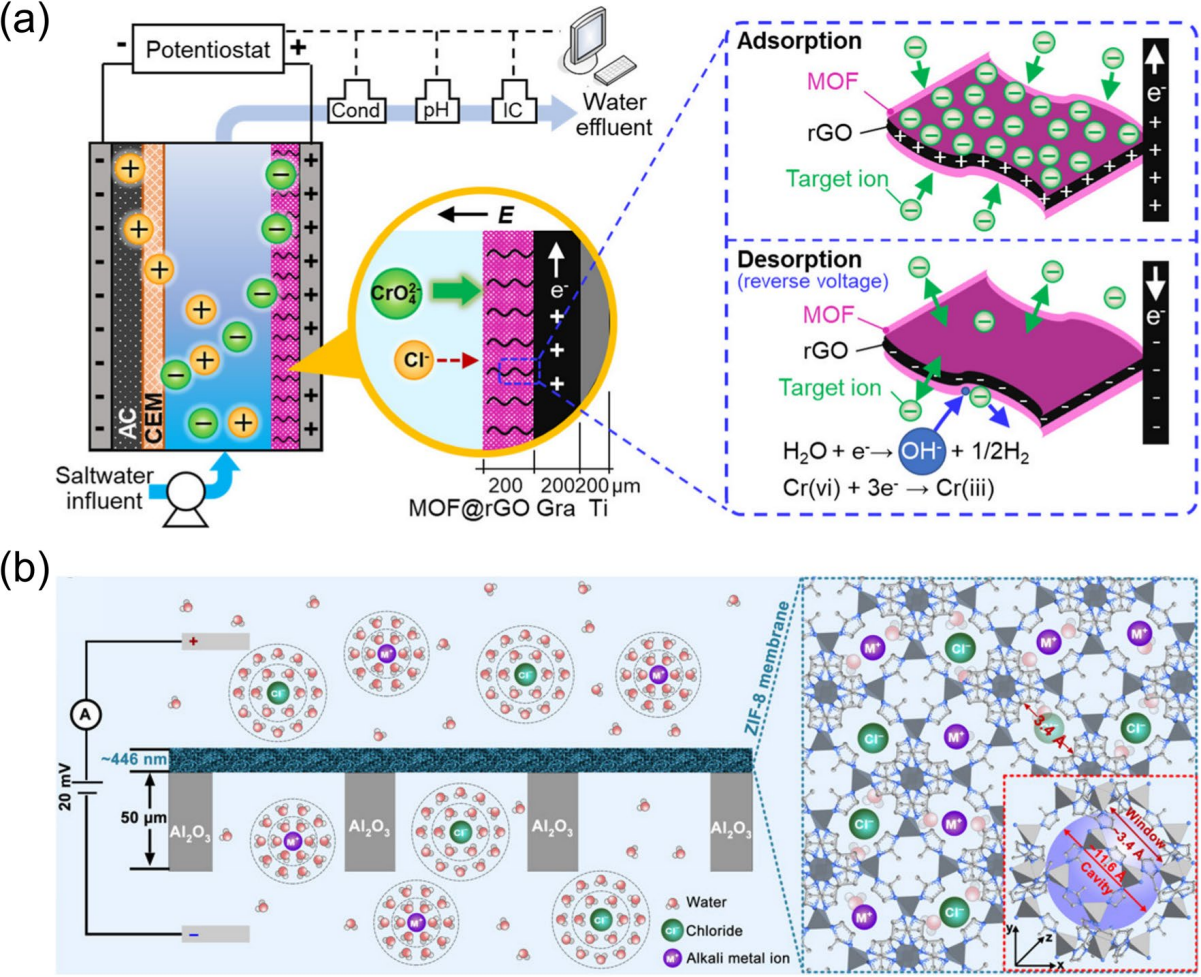
**a** Electrochemical adsorption and desorption of chromate using MOF-rGO-based electrode. Reprinted with permission from Ref. [78]. Copyright 2022 American Chemical Society. **b** A composite membrane of ZIF-8/GO/AAO being used under electric field for the selective ion transport. Reprinted with permission from Ref. [83]. Copyright 2018 The Authors, some rights reserved; exclusive licensee American Association for the Advancement of Science. Distributed under a Creative Commons Attribution NonCommercial License 4.0 (CC BY-NC), https://creativecommons.org/licenses/by-nc/4.0/

Kim et al*.* [77] demonstrated that the redox-active Cu-MOF-74 can be applied for the Faradaic electrosorption of Cd^2+^ from the simulated groundwater at a high capacity (> 0.9 mmol g^−1^) in an energy-efficient manner. Based on the experimental results, the selective adsorption of Cd^2+^ was attributed to the Cu(II)/Cu(I) reduction process, where the Cu-MOF-74-based electrode showed good regeneration ability and structural stability with minimal copper leaching (< 1 wt%). In a recent study, Zuo et al*.* [78] designed a hybrid adsorbent using reduced graphene oxide (rGO) and a cobalt-based MOF (Co-MOF). Endowed by the high electric conductivity of rGO as well as the strong and selective adsorption of CrO_4_^2−^ by the Co-MOF, this hybrid adsorbent can be employed as the anode to selectively and efficiently remove the toxic hexavalent chromium Cr(VI) (Fig. 4a). In addition, simply reversing the applied voltage can simultaneously reduce the adsorbed Cr(VI) to less toxic species, release the Cr species, and regenerate the adsorption sites in the Co-MOF. Inspired by the biological protein ion channels, Ma et al*.* [79] designed a novel electrode using UiO-66 with caged chorine ion-imprinted conductive polymers, where the caged polymers can adjust the pore dimensions of UiO-66 and offer molecular-scale channels for the transport of ions and electrons. As a result, the as-prepared UiO-66-based electrode can effectively separate chloride ions from the wastewater, where a number of merits were observed, including large chloride ion exchange capacity, fast adsorption, high selectivity as well as superior electrochemical durability. Through the in situ CuO-HKUST-1 conversion, Wang et al. [80] designed an electroactive polypyrrole (PPy)/HKUST-1 film composite with the MOFs anchored in the network of PPy nanorods for the selective extraction of Li^+^ ions from dilute resources. In particular, this PPy/HKUST-1 film exhibited a strong affinity for Li^+^ ions, which leads to an adsorption capacity of 37.55 mg g^−1^ while keeping the adsorption equilibrium time under 25 min. Moreover, the PPy/HKUST-1 film also showed significant selectivity against other ions (*e.g.*, K^+^, Mg^2+^, and Na^+^), which makes this film composite promising for lithium recovery from dilute solutions of different types.

Guo et al. [81] developed an HKUST-1-based membrane where the MOF crystals were threaded with polystyrene sulfonate (PSS), which not only enhanced the MOF’s stability in water but also provided sulfonic networks for selective ion transport. Compared with the pristine HKUST-1 membrane, the PSS threaded HKUST-1 membrane can improve the Li^+^ conductivity by five orders from 3.799 $\times$ 10^–9^ to 5.53 $\times$ 10^–4^ S cm^−1^ at 25 °C, meanwhile offering high selectivity over Mg^2+^, K^+^, and Na^+^ by leveraging the differences in the hydrated diameters of ions and their affinities to the sulfonate groups. Similarly, Xia et al. [76] recently designed a ZIF-based membrane functionalized with sulfonic groups, which exhibited strong hydrophilicity and selective transport of Cl^−^ over PO_4_^3−^, SO_4_^2−^, and Br^−^, primarily attributed to synergistically enhanced size-sieving effects and electrostatic interactions. Functionalization with the sulfonic acid groups can also be applied to UiO-66 to produce UiO-66-SO_3_H membranes with sulfonated channels at the angstrom scale, which can be used to achieve a threefold enhancement in cation permeation as well as improved selectivity of Na^+^ over Mg^2+^ [82]. In addition to the polymer supports, anodic aluminum oxide (AAO) can also be used as a support to fabricate MOFs-based membranes [83]. For instance, Zhang et al. [83] prepared a ZIF-8/GO/AAO composite membrane, where the interfacial growth of ZIF-8 crystals was facilitated with the nanoporous GO, which resulted in uniform ZIF-8/GO nanosheets on the AAO support. When the resultant ZIF-8/GO/AAO composite membrane was used under bias (Fig. 4b), it showed effective performance in selectively transporting Li^+^ in the presence of interfering metal ions (*e.g.*, Rb^+^, K^+^, and Na^+^).

**Fig. 5:**
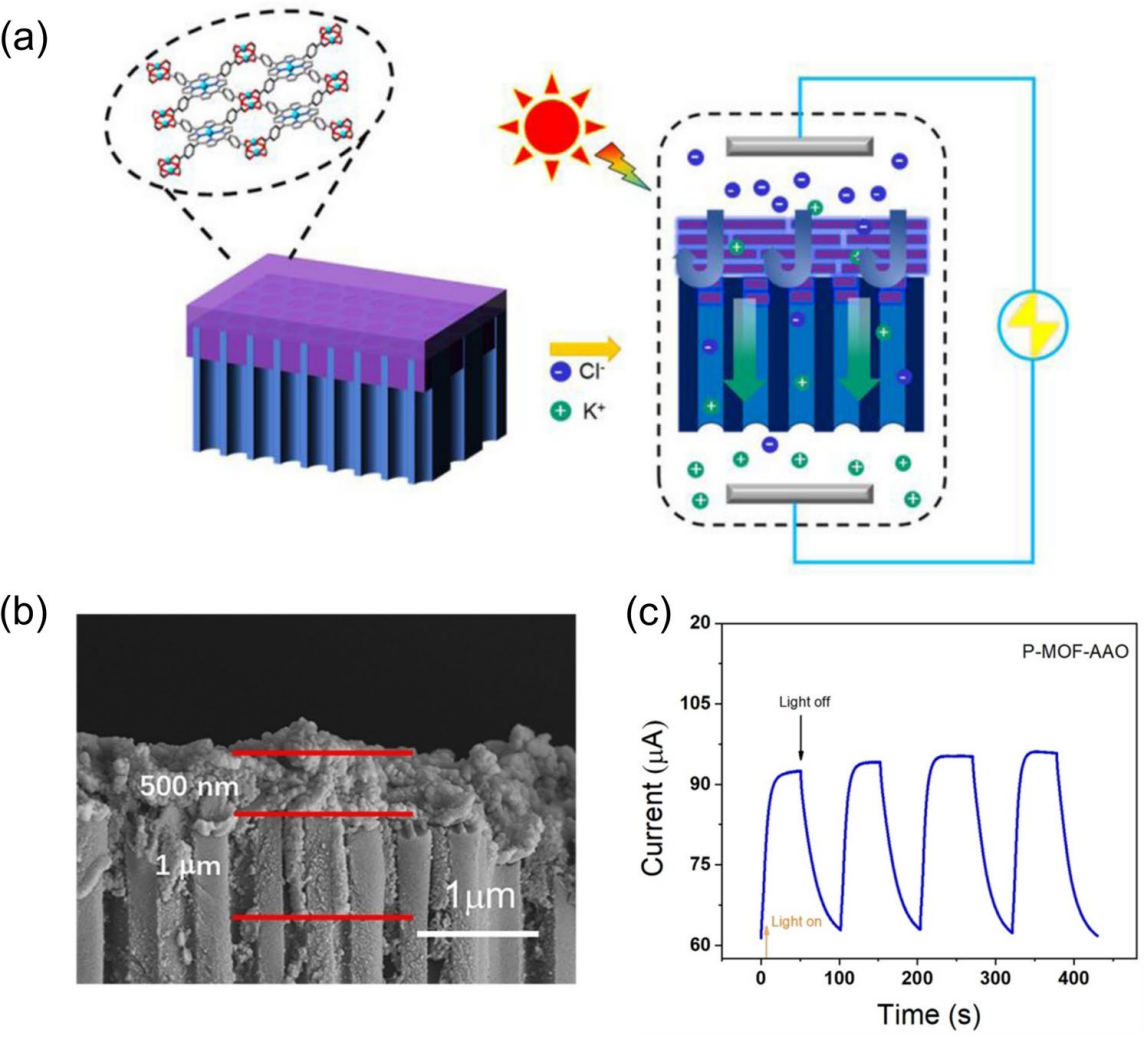
**a** Scheme of a composite membrane made of porphyrin MOF and AAO substrate, which is being used in the electrochemical system for osmotic energy conversion; **b** SEM image of the composite membrane; **c** Light-controlled ion current. Reprinted with permission from Ref. [86]. Copyright 2022 John Wiley and Sons

Recently, Wang et al. [84] constructed a bio-inspired, hybrid membrane via the covalent interactions between porous anodic aluminum and two-dimensional MOFs, which offer structural/chemical/charge heterogeneities that can promote asymmetric ion transport and ion selectivity, making it promising for effective water desalination. In addition, this hybrid membrane can be assembled into a power-conversion device to provide up to 1.6 W m^−2^ of power density when driven by a salinity gradient. Similarly, Liu et al. [75] designed a heterogeneous membrane using a positively charged, hydrophilic UiO-66-NH_2_ layer and the porous alumina membrane support, and the resulting heterogeneous membrane can be leveraged as selective ionic conductors for osmotic power harvesting to achieve a power density as high as 26.8 W m^−2^. Several approaches can be applied to further enhance the osmotic conversion efficiency, such as local temperature difference [85] and light irradiation [86]. For instance, Li et al. [86] applied cathodic deposition to fabricate porphyrin MOF on the porous substrate of anodic aluminum oxide, the composite of which offers a unique nanoporous structure as well as abundant carboxyl groups, allowing for highly selective ion permeability that leads to a high power generation from the salinity gradient (Fig. 5). Since the porphyrin linkers of the MOF are photoactive, the simulated sunlight irradiation can excite the ground-state electrons and subsequently increase the negative surface charge, which can stabilize the dehydrated cations, facilitate the ion transport with reduced activation energy, and eventually boost the power density by ~ 24%.

Overall, the studies mentioned above made encouraging demonstrations that the adjustable angstrom-scale pore windows, nano-scale cavities, and tunable surface functional groups of MOFs allow the modulated molecular sieve effect and surface charge to achieve the selective and effective separation of ionic species (*e.g.*, Cd^2+^, CrO_4_^2−^, and Li^+^), which offers new electrochemical strategies, where MOFs can be applied to remediate water contaminated with toxic ions, recover resources from wastewater, harvest lithium from brines, and achieve energy conversion via salinity gradient. In particular, MOFs can be fabricated into two different forms, either as redox-active electrodes for electrosorption or membranes for ion transport. When integrated as electrodes, MOFs can take advantage of the redox-active components in the framework for the selective Faradaic electrosorption of ions. On the other hand, MOFs can be integrated with polymers and porous metal oxide support (*e.g.*, AAO), where the MOF channel dimensions and properties can offer preferable size exclusion effects and electrostatic interactions to achieve selective ion transport under the electric field. As mentioned in the above demonstrations, these MOF-based membranes have been showing outstanding performance in extracting Li^+^ ions and energy harvesting from the salinity gradient. Due to the comparable dimensions of the hydrated ions and window sizes of MOFs, the ion transport through the MOF-based membranes is expected to be composed of multiple dehydration/hydration processes [83], where the atomic structures of solvated ions and the associated hydration energies need to be resolved in order to fully understand the ionic transport behaviors. A couple of strategies have also been verified to further improve ion transport and reduce the activation energy, such as light irradiation on the photoactive MOFs, which can generate negative surface charges for the stabilization of dehydrated cations.

## Challenges and Opportunities

The highly porous scaffolds, intriguing properties, and structural versatilities have granted MOFs superior performances in electrochemical water applications as illustrated in Sect. 2. Meanwhile, the unique, precise frameworks enabled by diverse metal–linker coordination environments also generate new research challenges and opportunities, such as understanding the electronic structures, resolving the nanoconfined structures and interactions, enhancing the stability and conductivity of MOFs, and probing structures at the atomic levels. Discussions and perspectives regarding these challenges and opportunities are laid out in the following sections.

### Electronic Structures

The electronic structure of MOFs plays an essential role in chemical bonding, structural integrity, chemical reactivity, light absorption property, as well as charge transfer capability [87–89]. The catalytic reactivity can be interpreted through frontier orbital compositions [90]. One of the active research focuses in the MOFs field is to determine their electronic structures [91], which results from the complex interplay among the metal nodes, organic ligands, and the corresponding metal–ligand interfaces [87]. The electronic structures of MOFs feature a discrete nature with more localized lowest unoccupied and highest occupied crystal orbitals [89].

**Fig. 6:**
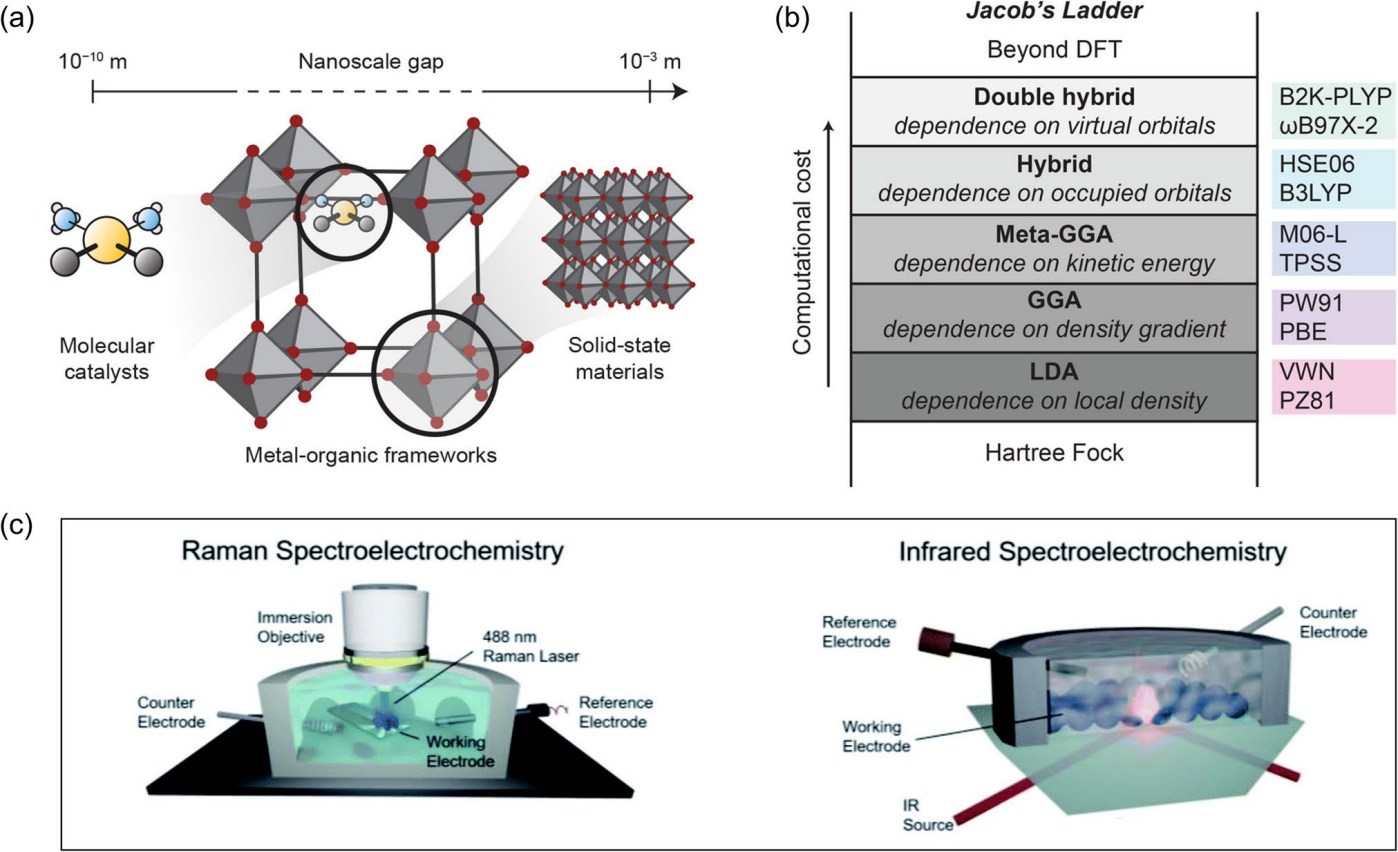
**a** Scheme of MOFs at different scales, leading to behaviors of both extended solids and molecules. **b** Jacob’s ladder of functionals. **a** and **b** are reprinted with permission from Ref. [91]. Copyright 2020 American Chemical Society. **c** Scheme of setup for the Raman and infrared spectroelectrochemistry. Reprinted from Ref. [94]. Copyright 2021 The Authors. Published by the Royal Society of Chemistry. Distributed under a Creative Commons Attribution 3.0 Unported (CC BY 3.0), https://creativecommons.org/licenses/by/3.0/

In order to gain full control of the stability and efficiency of MOFs-based functional materials in the electrochemical water remediation, delicate considerations are required for different aspects of MOFs’ electronic structures [92], such as band edge, band gap, energy level alignment, and electron localization. The electronic structures and charge transfer behaviors of MOFs can be investigated through a range of methods, such as density functional theory (DFT) simulations [88, 91–93], spectroelectrochemical measurements [94, 95], and transient absorption spectroscopy [96]. In particular, DFT-based electronic structure modeling offers rapid property prediction without facing synthetic challenges [91]. Despite MOFs’ duality in discrete molecules and extended solids (Fig. 6a), modeling has been demonstrated successful using either molecular or solid-state computational packages [91]. In comparison, DFT modeling (Fig. 6b) with MOF clusters is preferable when the subtle local changes play a more important role than the bulk stabilization effects and can leverage higher levels of theory; meanwhile, DFT modeling using extended MOF structures recognizes the dependence of delocalized molecular orbital energies on crystallographic-based electronic interactions [91]. The nature of charge transfer and active sites within MOFs can be probed through the distinct spectroscopic features using the in situ spectroelectrochemical approaches (Fig. 6c) [94, 97, 98]. These spectroscopic features at a particular applied potential can serve as signatures to identify the redox-accessible states. Furthermore, vibrational spectroscopy (*e.g.*, IR absorption and Raman scattering) can offer additional atomistic-level information by probing the molecular vibrations interacting with light with specific frequencies [98]. These aforementioned techniques can provide in-depth insights into MOFs’ electronic structures and charge transfer behaviors, which are of critical importance to both the fundamental understanding of the physical and chemical properties (*e.g.*, charge transfer within the three-dimensional coordination space) as well as the design principles for the multifunctional applications [87].

To optimize MOFs for improved efficiency during the electrochemical processes, it is crucial to achieve the targeted modulation of the electronic structures of MOFs, which can be accomplished through the modification to the building blocks of MOFs (*i.e.*, metal nodes and organic linkers) [87]. The density of electronic states can be tuned via several approaches [99], such as introducing secondary metals and/or linkers. In general, partial metal substitution affects MOFs’ band structures in terms of the relative/absolute energy positions [92]. For example, Dolgopolova et al*.* [99] examined the electronic properties of bimetallic MOFs with three distinct configurations, where the second metal sites were incorporated via either metal replacement, node extension, or ligand coordination. These isostructural bimetallic systems were tested and demonstrated that the engineering at the metal nodes could alter MOFs’ electronic structures by influencing the density of states adjacent to the Fermi edge, which could subsequently change their conductivity properties. During the metal substitutions, special considerations have to be given to several aspects, including but not limited to electron affinity, energy level alignment, dopant species, and dopant concentrations [92]. The modulation of the electronic structures of MOFs via partial metal substitution can create opportunities to achieve better performances in electrochemical water applications. For example, compared with the monometallic MOF-74, the bimetallic MOF-74 containing both manganese and iron demonstrated superior performance in degrading antibiotic sulfamethoxazole through the electro-Fenton process, which was largely attributed to the facilitated charge transfer between two metal sites [100]. Similarly, another bimetallic MOF was synthesized using Ni^2+^, Ce^3+^, and 1,3,5-benzenetricarboxylic acid, and with an optimized Ni^2+^/Ce^3+^ ratio, the resultant bimetallic MOF outperforms the monometallic counterpart in the electrochemical sensing of bisphenol A in water [101].

**Fig. 7:**
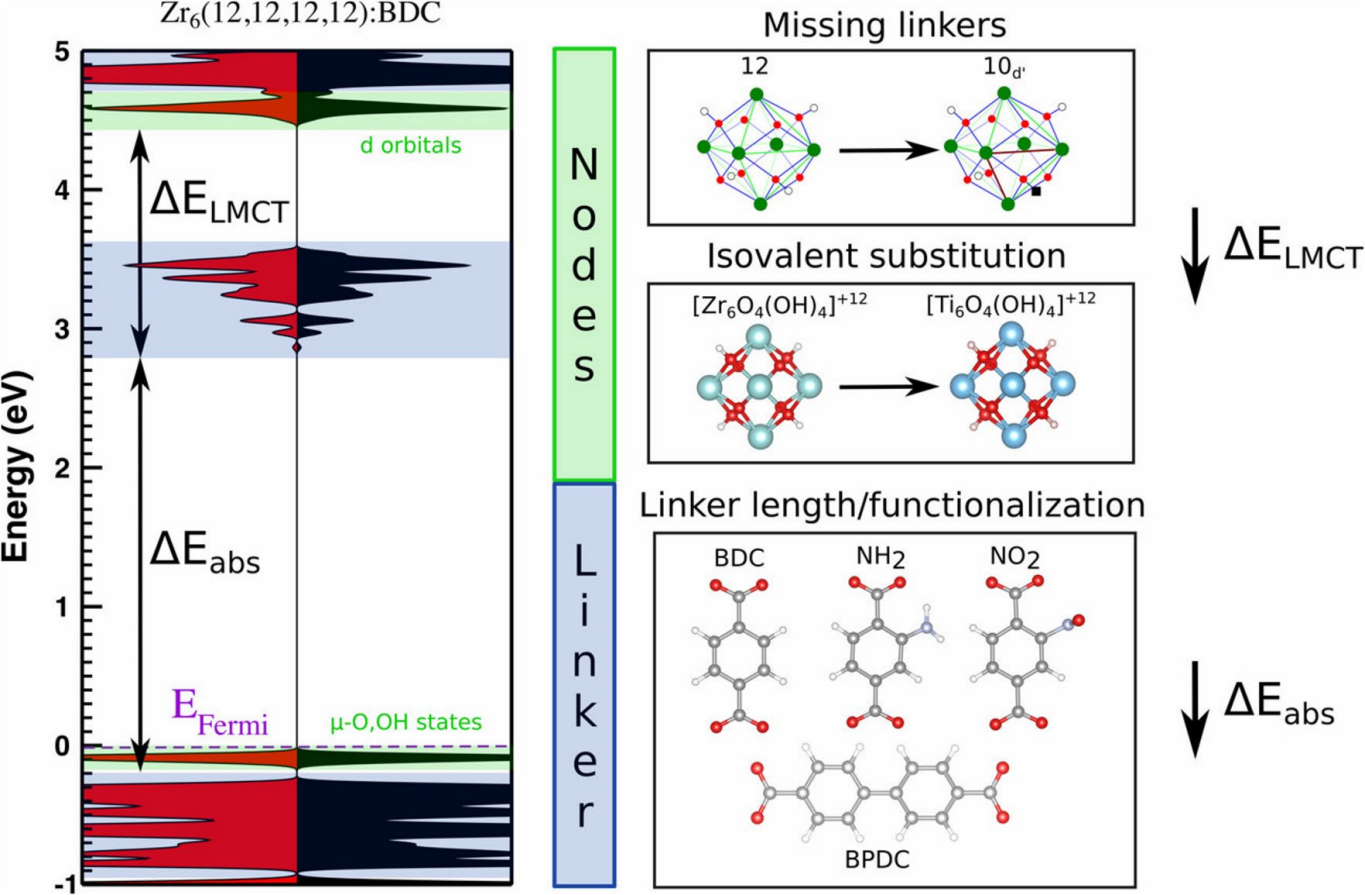
Alternation of UiO-66(Zr)’s electronic structure through structural modification. Reprinted with permission from Ref. [89]. Copyright 2017 American Chemical Society. Published under an ACS AuthorChoice License

Another alternative pathway to adjust the electronic structures of MOFs is to engineer the structural defects [89, 94]. A recent computational study by De Vos et al*.* (Fig. 7) [89] investigated how the UiO-66’s electronic structure can be tuned by the missing-linker defects, which demonstrated that the partial removal of the linkers changes the local Zr environment by lowering the unoccupied d orbitals; meanwhile, minimal changes were observed regarding the states of organic linkers. The results also suggested the central role of local distortions at the constituent nodes, which is capable of resulting in the highest variation in the electronic structure. In addition to the missing-linker defects, unsaturated defects can also be incorporated into MOFs with the presence of secondary linkers that lack binding functional groups, which can eventually create coordinatively unsaturated active sites after synthesis [94]. As demonstrated by Heidary et al*.* [94], defective Ni-MOF-74 was synthesized with nickel ions and a ligand mixture of 2,5-dihydroxyterephthalic acid and 2-hydroxyterephthalic acid, which exhibited a unique Ni local environment due to the unsaturated coordination and presented distinct interactions with guest molecules.

The aforementioned examples have highlighted MOFs’ electronic structures from different aspects, including the essential roles, characterization approaches, and design principles (*e.g.*, metal addition/substitution, linker modification, and defects engineering). Considering the significance of MOFs’ electronic structures in the structural integrity, intrinsic properties, as well as their performances in electrochemical water remediations, further research efforts are still needed to gain a complete understanding of the electronic structures with the aid of advanced tools (*e.g.*, soft X-ray spectroscopy), especially under the *operando* conditions [102], which will eventually allow more precise and targeted MOFs design to obtain optimized electrochemical water remediation (*e.g.*, stability, reactivity, and selectivity).

### Nanoconfinement

Due to the angstrom-scale and precisely controlled pore structures of MOFs, the three-dimensional scaffolds can pose particular nanoconfined effects on guest nanoparticles [103, 104] and molecules (*e.g.*, organic dyes [105] and water [106–108]), which results in distinct guest–host interactions, leading to some particular chemical and physical phenomena that are unachievable with the bulk condition, such as variable spatial orientation, unique phase properties, and uncommon thermodynamic behaviors [109, 110].

**Fig. 8:**
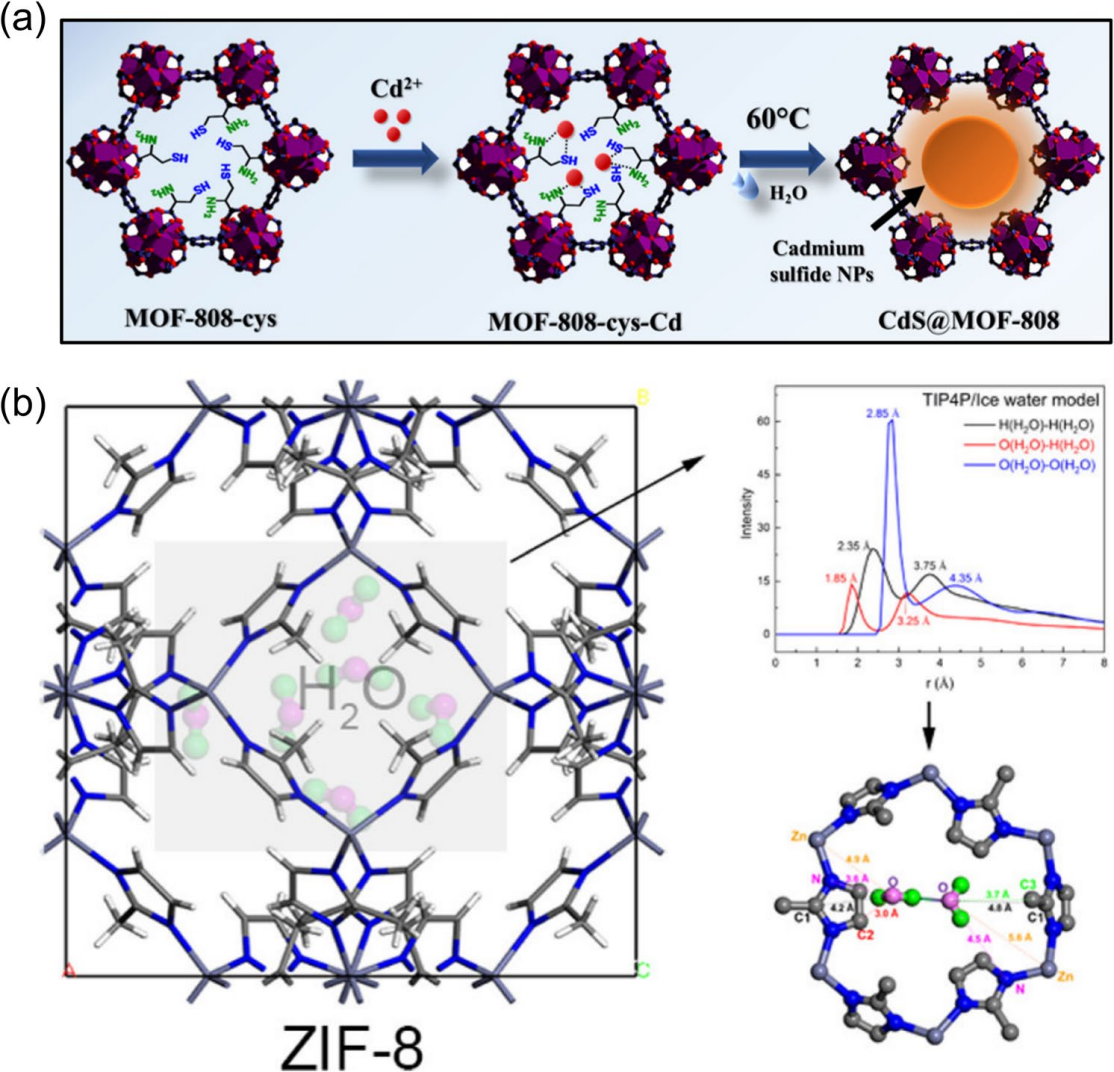
**a** Schematic illustration of growing confined CdS nanoparticles within the pores of MOF-808. Reprinted with permission from Ref. [109]. Copyright 2022 American Chemical Society. **b** Water molecules being confined in the nanopores of ZIF-8. Reprinted with permission from Ref. [107]. Copyright 2021 The Authors. Published by American Chemical Society. Distributed under a Creative Commons Attribution-NonCommercial-NoDerivatives 4.0 International (CC BY-NC-ND 4.0), https://creativecommons.org/licenses/by-nc-nd/4.0/

There are generally two approaches to encapsulate nanoparticles within the coordination networks of MOFs: (1) synthesis of MOFs in the presence of pre-prepared nanoparticles and (2) incorporation of nanoparticle precursors followed by the conversion within MOFs [104]. For instance, Xu et al. [111] injected pre-prepared, poly(vinylpyrrolidone)-stabilized suspensions of platinum nanoparticles into the UiO-66 precursors, and after the hydrothermal synthesis procedures, Pt@UiO-66 heterostructures were successfully obtained, offering a highly selective and sensitive platform to detect hydrogen peroxide. In addition to growing MOF matrices around the pre-prepared nanoparticles, the MOF/nanoparticle composite can also be fabricated via the post-synthetic approach. Taking a recent work by Ghosh et al*.* [109] for example, 1–2 nm CdS nanoparticles were grown inside MOF-808 with the aid of L-cysteine anchored through the post-synthetic ligand exchange (Fig. 8a). Confined within MOFs pores, the CdS nanoparticles were placed in close proximity to Zr^4+^ clusters, which stabilized the charge-separated states and subsequently boosted the electrocatalytic activity by ~ 69 times compared with the unconfined CdS nanoparticles. The enhanced performance mainly stemmed from the efficient and fast electron transfer kinetics of CdS provided by MOF-808’s confined environment. In addition to nanoparticles, molecules can also be encapsulated within the confined environments of MOFs to achieve exceptional performances [105]. For instance, Let et al. [105] applied an ion exchange approach to incorporate the Rhodamine B (*i.e.*, a cationic dye) into an anionic MOF, where the confinement inhibited molecular aggregation and the subsequent luminescence quenching, making the composite system a promising platform for Fe^3+^ detection. When molecules are restrained within pore dimensions at comparable scales, complex molecule–molecule and molecule–surface interactions come into play, which gives rise to spatial rearrangement [110, 112]. For example, as ions enter the angstrom-scale pores, the solvated ions need to go through a dehydration process, which has a strong dependence on the hydration energy, resulting in different energy barriers (*i.e.*, enthalpy for ion dehydration) and selectivities for ion transport [113, 114]. In a recent study by Li et al*.* [114], the ZIF-90 layer was fabricated on a porous metal oxide membrane, which introduced angstrom-scale pore channels to the membrane system. As demonstrated through the molecular dynamics simulations, the pore-confined ions were dehydrated to a certain degree as a result of the ionic interactions as well as the structural confinement, which led to variations in the effective ionic diameters (*e.g.*, d(K^+^) > d(Na^+^) > d(Li^+^)) and energy barriers for ion transport.

Last but not least, MOFs’ confined environments can also modify the water behaviors [106–108]. In particular, the presence of heterogeneous polar/nonpolar domains can influence the hydrogen-bond networks [108]. For instance, the molecular dynamics simulations carried out by Zhang et al. [107] suggested the formation of water dimers in ZIF-8 pores (Fig. 8b), where weak hydrogen bonds were observed between water dimers and the terminal functional groups of the imidazole ligands (*i.e.*, CH and methyl groups). In addition, Medders et al. [108] applied computational infrared spectroscopy to assess water orientational dynamics, which showed that the competitive interactions among water molecules and the frameworks within the confined environment of MIL-53 (Cr) could result in substantially retarded hydrogen bond dynamics as compared to that in the bulk condition. With the aid of the first-principles molecular dynamics simulations, Haigis et al. [106] compared structures and dynamics of water confined in two different phases of the flexible MIL-53(Cr) (*i.e.*, narrow-pore phase *vs.* large-pore phase), where results exhibited more disordered hydration and considerably slower water reorientation dynamics in the large-pore phase. The variation in the confined water structures has direct impacts on a variety of applications, such as proton transport [115–117]. In a recent computational study, Vu et al. [115] carried out density functional theory investigations on the equivalent water clusters in MOF-801 cavities (*i.e.*, dimer, pentamer, and octamer), which exhibited different barriers for the proton transfer, where the minimum barrier of 16 kJ mol^−1^ was found in the case of pentamer cluster.

The investigations mentioned above provide a clear demonstration that the angstrom-level, well-designed architectures of MOFs can induce distinct nanoconfined effects on various elements (*i.e.*, nanoparticles and molecules), which could give rise to unique physical and chemical phenomena (*e.g.*, spatial arrangement and reaction kinetics). The nanoconfined environments from MOFs’ architectures can be feasibly tailored by adjusting the surface chemistry, morphologies, or pore dimensions [110]. In another example related to the spatial orientation of confined water molecules, owing to the feasible formation of the hydrogen bonds between water molecules and the frameworks, the *μ*_2_-OH positions within MOFs can be tuned to affect the water microstructure [108]. Despite the growing understanding of the distinct confined effects, future research efforts are still needed to make the connections between the nano-confined environments (*e.g.*, local geometry and chemistry) and the resulting outcomes (*e.g.*, spatial orientation, diffusion behaviors, and reaction kinetics). Such information can then be leveraged to achieve environmental significance in electrochemical water remediation.

### Electrochemical Stability of MOFs

**Fig. 9:**
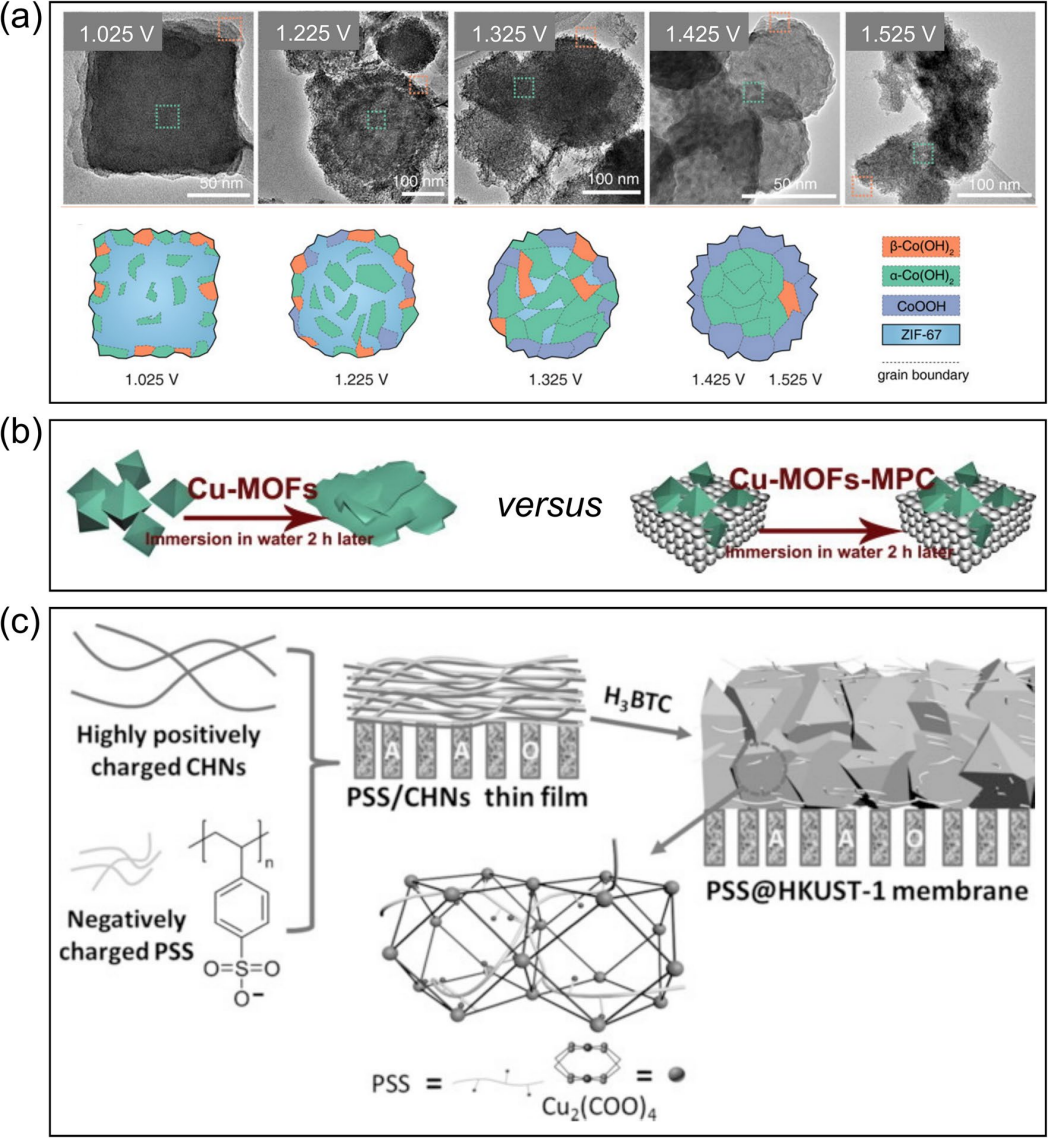
**a** Structural evolution of ZIF-67 as a function of applied potentials after 1-h amperometric treatments. Adapted with permission from Ref. [119]. Copyright 2020 American Chemical Society. **b** A scheme illustrating that the microporous carbon can protect Cu-based MOFs against water. Reprinted from Ref. [118], Copyright (2014), with permission from Elsevier. **c** A schematic procedure to manufacture a composite MOF-based membrane using polystyrene sulfonate (PSS) to obtain better stability in water. Reprinted with permission from Ref. [81]. Copyright 2016 Wiley‐VCH Verlag GmbH & Co. KGaA, Weinheim

The diverse properties of MOFs are enabled by their chemical modularity, which, on the other hand, poses challenges to MOFs’ stability under different operating conditions. In particular, MOF structures are held intact primarily through the coordinate bonds potentially with several types of weak interactions (*e.g.*, π–π stacking), which could result in structural flexibility or instability during electrochemical processes [118]. For instance, the overcharging process could potentially degrade the metal nodes into metal oxides or zero-valent metals [77]. In a recent investigation, Zheng et al. [119] inspected the electrochemical stability of the zeolitic imidazolate framework-67 (ZIF-67) using operando spectroelectrochemistry, which illustrated how amperometry and cyclic voltammetry affected ZIF-67’s structures and morphologies. More specifically, electrochemical treatments could convert ZIF-67 to *α/β*-Co(OH)_2_ and CoOOH (Fig. 9a) which dominated oxygen evolution reactions.

The improvement in the water and electrochemical stability of MOFs will contribute to the transformative development of MOFs-based electrochemical water remediation processes. Various strategies have been proven successful to enhance the stability of MOFs during electrochemical water remediation. In general, higher aqueous stability can be achieved with the use of shorter linkers, higher-valence metals, and higher metal-linker connectivity [74, 120]. For instance, Zhou et al. [121] deposited Mn-PCN-222, a manganese metalloporphyrin film, onto indium tin oxide (ITO) to create a voltammetric sensor for the highly sensitive detection of a variety of trace pollutants, including but not limited to heavy metal ions and phenolic toxins. Owing to the strong porphyrin–Mn^III^ chelation and robust Zr_6_ clusters, the as-prepared MOF-based sensor exhibited excellent electrochemical stability during multi-cycle measurements with minimal changes to the molecular structures. In addition, the usage of hydrophobic/inert ligands as the building units of MOFs, such as imidazolate and pyrazolate, can effectively avoid nucleophilic attack from the water [122, 123]. Lopa et al. [122] designed a glucose sensor by integrating the glassy carbon electrode with metal azolate framework composed of Co^II^ and 2-methylimidazole, which demonstrated long-term stability for glucose sensing. Nevertheless, the long-time and overcharging electrochemical treatments could still cause irreversible damage to the structures and morphologies of this type of MOFs by undermining the metal–ligand coordination [119]. Another approach to enhancing the stability of MOFs is to construct hybrid MOFs [120]. In a recent study, Shen et al. [120] applied modular synthesis to coordinate cobalt carboxylate clusters, a type of paddle-wheel and highly reactive clusters, into the Fe(III) dicarboxylate frameworks to form a hybrid MOF, which exhibited not only extraordinary electrochemical activity but also excellent stability in water or alkaline environments. In addition, other functional materials, such as macroporous carbon [118] and polystyrene sulfonate [81], can also be used to improve the stability of MOFs. For instance, Zhang et al. [118] designed a hybrid composite using macroporous carbon and a copper-based MOF (Fig. 9b), which was proven highly stable in water or phosphate buffer solution during electrochemical processes, making the hybrid composite a promising sensing platform for biomolecules. In another example, the membrane fabricated from HKUST-1 and polystyrene sulfonate (Fig. 9c) demonstrated extraordinary stability even after two-month immersion in water, where the polystyrene sulfonate successfully secured both the framework and the labile Cu^2+^ sites, showing significant potential to separate lithium ions from aqueous solutions [81].

Despite the aforementioned efforts, additional research is still imperative regarding MOFs’ thermodynamic stability in electrochemical environments. Particularly, when MOFs are used as active functional materials, it is essential to evaluate the structural stability to accurately establish the functionalities of MOFs [119, 124]. Meanwhile, the recent development of some advanced tools, such as artificial intelligence, should also be applied to screen stable candidates from the vast MOFs database for electrochemical water remediation. On the other hand, if the structural flexibility of MOFs can be leveraged to achieve controllable structural reconstruction, it could potentially generate new active catalysts with outstanding performance [125]. For example, Yao et al. [125] reconstructed a bismuth-based MOF (Bi-MOF) to Bi_2_O_2_CO_3_ via electrolyte mediation, which subsequently reduced to Bi through potential regulation, offering unsaturated active sites and exceptional performance in formate production. Once again, it highlighted the critical importance of monitoring the structural evolution of MOFs during electrochemical processes.

### Conductivity in MOF Systems

Most MOFs are electronically insulating, which can be primarily attributed to the high electronegativity of the predominantly used carboxylate ligand’s oxygen atoms, causing inadequate interactions with metal *d* orbitals and subsequently limiting the electrical conductivity [126, 127]. Broadly, the typical binding between the hard atoms (*e.g.*, nitrogen and oxygen) of the redox-inactive linkers and hard metal ions results in the lack of free charge carriers nor feasible charge transfer pathways, making the conductivity of vast MOFs less than 10^–10^ S cm^−1^ [128]. The poor conductivity of most MOFs is another major factor that limits their capabilities of electrochemical water remediation (*e.g.*, electro-Fenton processes [51]) and other electrochemical processes (*e.g.*, energy storage [129] and energy conversion [130]).

**Fig. 10:**
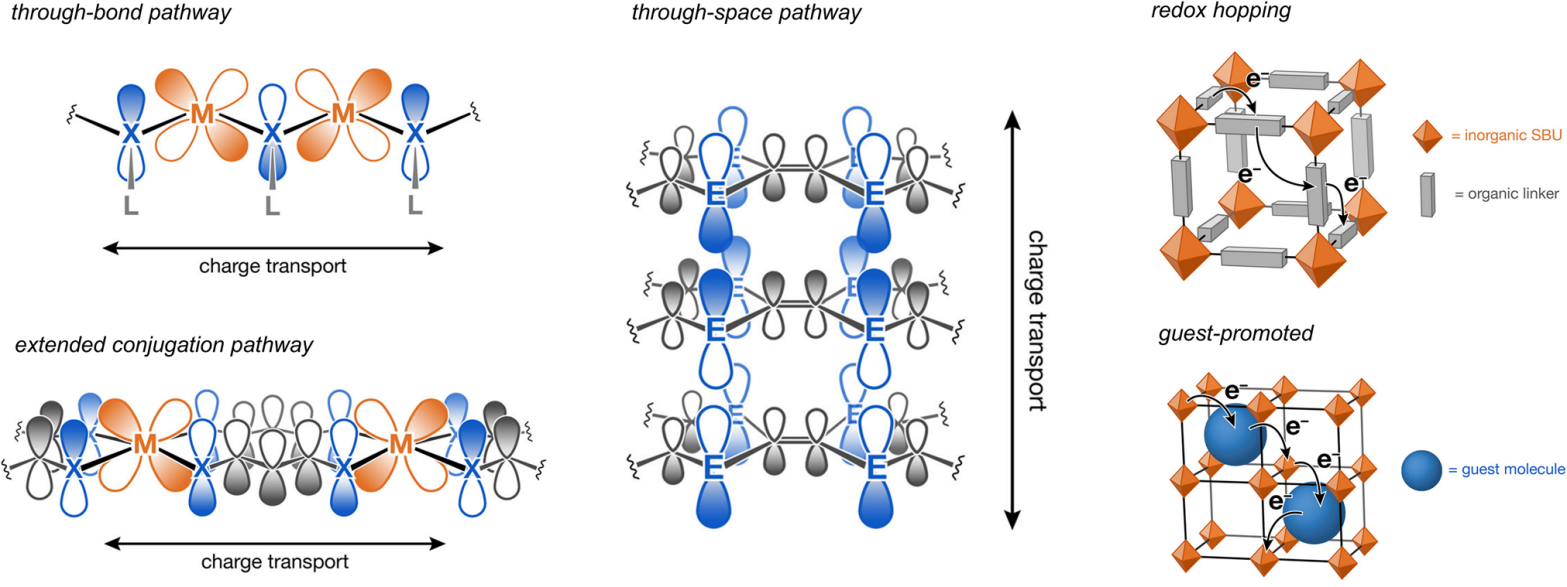
Schematic illustrations of the design strategies to improve the electrical conductivities of MOFs. Adapted with permission from Ref. [132]. Copyright 2020 American Chemical Society. Published under a Creative Commons Attribution (CC BY) License

Recently, several strategies have been developed to overcome the conductivity limitations of MOFs, which has produced many electrically conductive MOFs [126, 128, 131-134]. In general, charge transfer within MOFs can occur in two different modes, including band transport and hopping transport [128, 132, 135]. The conductivity of MOFs can be improved either by enhancing the metal-linker electronic coupling which helps to boost the band dispersion or by introducing impurity (*e.g.*, doping) and defects (*e.g.*, vacancies) which shifts the Fermi level and raises the concentrations of charge carriers in the band gap [7]. Typically, there are several design strategies that can be applied to enhance MOFs’ electrical conductivities, such as through-bond(/space) pathways, the extended conjugation pathway, and redox hopping (Fig. 10) [132]. Taking the two-dimensional MOFs for example, the π-conjugated organic linkers can induce substantial orbital interactions with the metal sites, which can significantly boost electrical conductivity [136]. In addition to these in-plane orbital interactions, the perpendicular charge transport across the two-dimensional layers can also contribute to MOFs’ improved electrical conductivity [137]. Judicious selection of packing motif and metal cation of the two-dimensional MOFs could be used to modulate the absolute conductivity [131]. The construction of metal-sulfur chains or planes can also bring profound orbital interactions through metal *d* orbitals and sulfur *p* orbitals, which can achieve enhanced charge transport as well [126]. Moreover, increasing the density of charge carriers (*e.g.*, the use of metal ions with unpaired high-energy electrons or stable organic radicals) can also promote electrical conductivity [128].

Incorporating other functional materials/molecules with MOFs is another promising route to enhance the electrical conductivity for electrochemical water remediation. For example, molecular infiltration has been tested, which typically coordinates redox-active, conjugated molecules with the framework and forms chains to facilitate electrical conductivity [134, 138, 139]. Similar to molecules, encapsulated conductive polymers can also improve the electrical conductivity of MOFs. For example, MIL-101(Cr) was used by Le Ouay et al. [140] to host conductive polymers (*i.e*., poly(3,4-ethylenedioxythiophene)), and the resultant composite exhibited a conductivity of 1.1 × 10^–3^ S cm^−1^ without sacrificing porosity. In general, the promoted conductivity of this type of polymer@MOFs composite can be rationalized via several factors: (1) highly oriented and ordered polymers, (2) polymer–ligand interactions, and (3) hole doping through iodine during polymerization [132]. In addition to the redox-active molecules and conductive polymers, the integration of MOFs with other functional materials (*e.g.*, GO, carbon nanotubes, and nanoparticles) can also improve the electrical conductivity and subsequently enhance the performance of the MOFs-based systems in the electrochemical water remediation, as exemplified in the previous sections.

The aforementioned research has offered some exciting advancements in the fundamental understanding and delicate control of the electrical conductivity of MOFs, which have enabled the simultaneous adoption of huge porosity, large surface area, and electronic delocalization, eventually contributing to the accelerated and widened implementation of MOFs in electrochemical water applications, such as sensing and catalytic processes.

### Atomic Structures of MOFs

Oftentimes, it is the local structures rather than the long-range order that determines the properties and drives the functionalities [141]. As one of MOFs’ key features, the heterogeneities in local structures (*e.g.*, oxidation-state incongruities and bonding misbehaviors) can lead to unexpected phenomena [142]. For instance, the increased oxidation level of irons in Fe(1,2,3-triazolate)_2_(BF_4_)_x_ creates low-spin, mixed-valence centers of irons (Fe^II^/Fe^III^), a heterogeneity that can promote intervalence charge transfer and improve the conductivity up to 0.3 S cm^−1^, which is eight orders of magnitude better than the counterpart without mixed valence [143]. In another study, mixed linkers of benzene-1,3,5-tricarboxylate (btc) and pyridine-3,5-dicarboxylate (pydc) were introduced into a Ru-based MOF, where the heterogeneity in linkers resulted in partial reduction of Ru, accompanied with more coordinatively unsaturated sites, which eventually altered the host–guest interactions as well as the catalytic ability of the Ru-based MOF [144]. These structural heterogeneities of MOFs play a critical role in electrochemical water remediation applications. For example, the size-sieving performance of MOFs could be undermined by the enlarged pore dimensions as a result of defects [74]. On the other hand, defect engineering can be applied to MOFs to achieve tunable ionic/proton conductivity by creating ligand and/or metal heterogeneities [145].

Despite their crucial importance, these subtle chemical aspects in MOF structures are usually understated in the literature, where MOFs are idealized by assuming structural rigidity [142]. In order to update the existing structure–property relationships by taking the structural deviations into consideration, it is imperative to obtain the accurate characterization of these local structures of MOFs, which remains a critical challenge that is unable to address through routine characterization methods. For example, conventional Bragg diffraction can only provide information about the average crystal structure [146]. In order to resolve MOFs’ local structures, advanced tools are necessary, such as pair distribution function (PDF) [141] and X-ray absorption spectroscopy (XAS) [147]. Among these advanced techniques, the PDF approach has been receiving increasing attention as an outstanding tool to investigate atomic structures over local-, medium-, and long-range scales [102, 148–152].

The PDF analysis can be carried out using different scattering probes, including electrons, neutrons, and X-rays, where X-rays are broadly deemed as the most versatile and accessible probe [150]. The procedures for a general PDF analysis of the X-ray scattering data generally involve three steps: (1) a total scattering measurement, (2) corrections of scattering intensity, and (3) the sine Fourier transform [148]. Briefly, high-energy X-rays are firstly scattered off the samples (*e.g.*, solution, crystalline, or amorphous materials) and then collected using a large 2D detector over a wide angular range [149]. After the total scattering measurements, accurate and comprehensive corrections must be applied to the scattering intensities by taking several essential factors into consideration, such as background scattering, Compton scattering, and X-ray polarization [148, 153]. Following the intensity corrections, the structure function (*i.e.*, S(Q)) can be attained and then subjected to the sine Fourier transform (Eqs. 7 and 8) [153]).

7 $$G\left(r\right)= \frac{2}{\pi }{\int }_{{Q}_{\mathrm{min}}}^{{Q}_{\mathrm{max}}}F\left(Q\right)sinQr \mathrm{d}Q$$

8 $$F\left(Q\right)=Q\times [S(Q)- 1]$$

where *G*(*r*) represents the pair distribution function describing the possibility of observing atomic pairs separated by a distance of *r* and *Q* denotes the magnitude of the scattering vector.

**Fig. 11:**
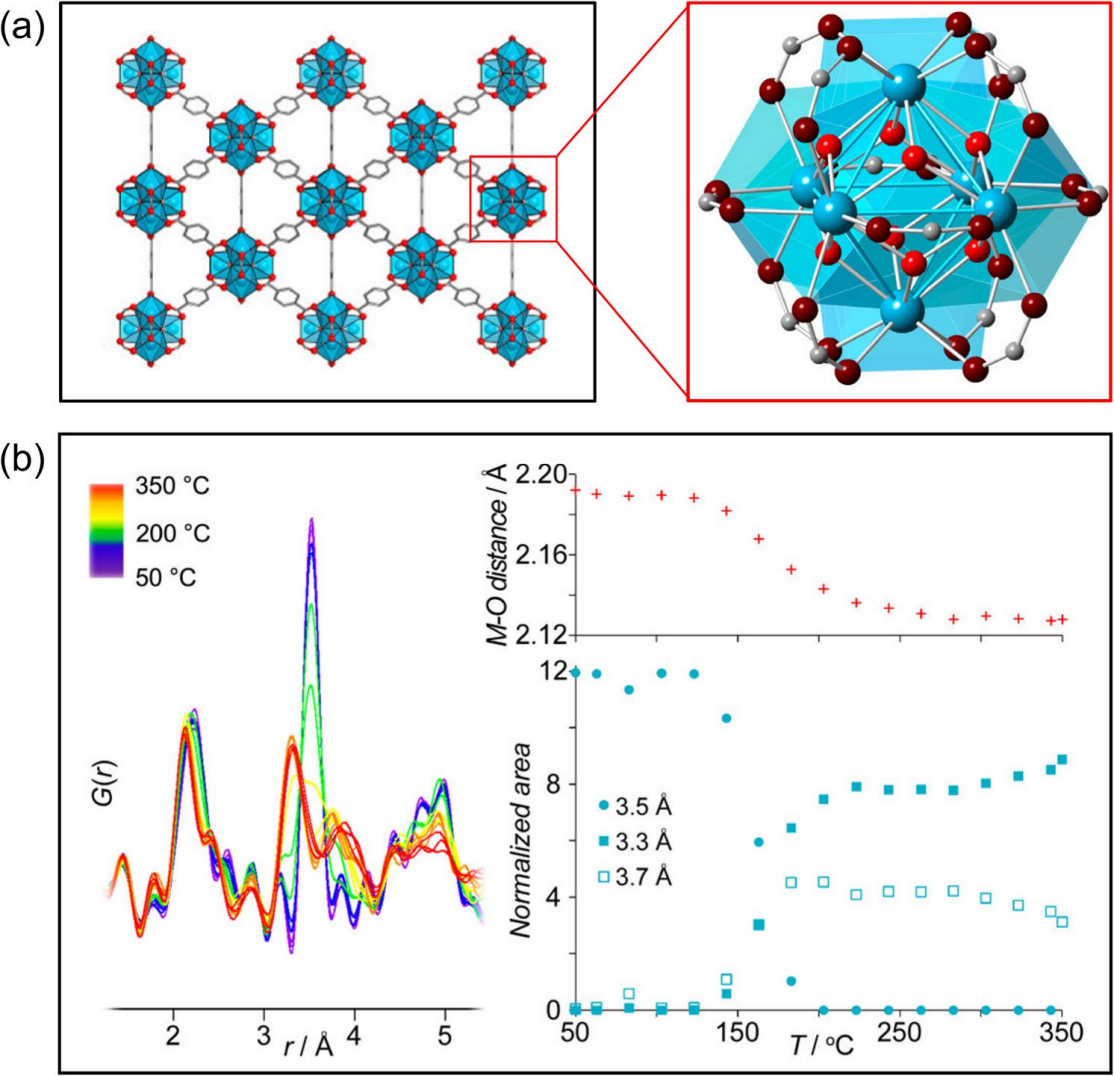
**a** The framework and M_6_(O)_8_ nodes of UiO-66. **b** PDFs of Zr-based UiO-66 as a function of temperatures. Adapted with permission from Ref. [164]. Copyright 2016 American Chemical Society

In recent years, PDF has been demonstrated as a decent tool to investigate the atomic structures of both bare MOFs [141, 154–156] and MOF–guest interactions [157–160], which have contributed to the fundamental understanding of MOFs in several aspects [161], including but not limited to MOF formation at the pre-crystalline stages [162], the nucleation and growth of MOFs [163], the solid-state phase transition [156], node distortions [164], and controlled capture/release of guest molecules [165]. For example, in a recent study, a zirconium–phosphonate-based MOF was put through a phase transition from the crystalline phase to a new semicrystalline–amorphous phase, which showed a threefold enhancement in the proton conductivity [156]. Through the PDF analysis, it was found that the phase transition did not affect the local structures (*i.e.*, < 4.5 Å) but changed the medium-/long-range order, which suggested the zirconium assemblies remained intact but ligands were shifted remarkably to achieve different orientations during the phase transition. In situ PDF can also be coupled with DFT computations to monitor the node distortions induced by temperature [164], where the results demonstrated that even mild temperatures (*i.e.*, < 150 °C) can alter MOF nodes regarding the local geometries (Fig. 11), suggesting flexible side of the metal clusters in MOFs. In addition to the atomic structures of bare MOFs, PDF approaches have also been widely applied to interpret the interactions between the host MOFs and the guest molecules. Taking the adsorption of I_2_ (*i.e.*, a radioactive fission product) by MFM-300 (*i.e.*, a robust MOF series, MFM: Manchester Framework Material) for example [160], the inclusion of the PDF analysis can synergistically complement the fundamental understanding of not only the intramolecular I–I correlations but also the intermolecular interactions among guest I_2_ molecules and host MFM-300. Moreover, Rangwani et al. [158] recently employed the differential PDF (dPDF) to study the interactions between ${\mathrm{Sb}(\mathrm{OH})}_{6}^{-}$ and the Zr_6_ node of NU-1000, which unveiled the ${\eta }_{2}{\mu }_{2}$ fashion of the interactions, shedding light on the practical engineering applications of water-stable MOFs for the adsorptive removal of hazards from water. In another work, NU-1000 was adopted as a highly porous support to host single/few-atom Pt clusters using atomic layer deposition [159], where PDF served as a critical tool to reveal the atomic correlations of Zr–Zr, Zr–Pt, and Pt–Pt pairs, which offered insights into the distinct local environment and host–guest interactions. As demonstrated by the above studies, the successful implementation of the PDF analysis has offered valuable atomic insights to elucidate the critically important relationships among MOFs’ structures, properties, and functions. It is expected that the PDF analysis will continue to serve as a crucial tool to decode the structure–property–function relations of MOFs under both in situ and ex situ conditions, which will eventually contribute to the practical development of MOFs with desired electrochemical functionalities for efficient water remediation.

## Conclusions and Outlook

Due to their unique structural and chemical tunability, MOFs have been studied extensively in a wide domain of electrochemical water applications (*i.e.*, reactions, sensing, and separations). In particular, the synergistically combined advantages of periodically extended solids and discrete molecules in MOFs endow specific selectivity toward the target molecules and facilitate the nanoconfined guest–host interactions to achieve amplified reactivities, allowing the sensitive detection of pollutants, efficient degradation of wastes, and selective resource/energy recoveries. To further unlock MOFs’ full potential, advancements of understanding in several fundamental aspects (*e.g.*, electronic structure, nanoconfinement, stability, conductivity, and atomic structure) are still needed to boost the performances of MOFs (*e.g.*, efficiency and selectivity), which will be achieved by taking advantages of several advanced tools, including but not limited to density functional theory, spectroelectrochemistry, and pair distribution function analysis. Some of these areas that are worth particular attention are listed as follows:

1. When applied in electrochemical water applications, MOFs are subjected to a range of different aqueous environments (*e.g.*, various pHs and temperatures). Due to their construction nature, MOFs may undergo substantial structural deviations, which would generate secondary pollution, such as metal leaching from MOFs. In addition, the structural deviation of MOFs could result in the formation of other functional materials (*e.g.*, metal oxides), which potentially predominate the electrochemical reactions. Therefore, in order to accurately resolve MOFs’ roles and avoid secondary contamination during electrochemical water remediation, it is critical to conduct comprehensive investigations of MOFs’ stabilities. In the meantime, computational efforts, such as molecular dynamics simulations and machine learning, can aid in prescreening of stable MOFs for electrochemical water applications.
2. The MOF-based electro-Fenton process has two rate-limiting steps: (1) H_2_O_2_ production and (2) Fe^2+^/Fe^3+^ circulation [51]. The improvement of MOF’s conductivity can promote electron transfer within the framework, which will subsequently enhance the Fe^2+^/Fe^3+^ cycle and provide more active sites for the activation of H_2_O_2_ to produce reactive radicals for the degradation of organic pollutants [166]. On the other hand, the improved conductivity of MOFs could also facilitate the oxygen reduction reaction to H_2_O instead of H_2_O_2_, especially for the iron-based MOFs [167], which poses detrimental effects on the efficiency of the electro-Fenton process. Therefore, to achieve the optimized electro-Fenton process, additional and deliberate considerations have to be given to the structural design of MOFs, such that the products of the oxygen reduction reaction can be selectively controlled.
3. As mentioned in Sect. 3.5, the PDF technique is emerging as a powerful tool in resolving local structures of MOFs, such as local distortions and guest–host interactions. To focus on the heterogeneities in MOF structures, the differential PDF analysis is frequently used, which can be obtained through the subtraction of the PDF of the heterogeneous structure from that of the reference structure [161]. The short-range heterogeneities can also be extracted from PDF using a range of modeling approaches, such as small-box modeling and reverse Monte Carlo (RMC) modeling [150]. For example, small-box modeling can be applied to periodic structures, which can derive local deviations based on real-space information [168], while RMC modeling works well for structures lacking long-range orderings. The effectiveness and feasibility of the fitting depend heavily on the signal-to-noise ratio of the PDF data. In order to extract reliable and precise structural information at the short-range scale, it is of crucial importance to minimize the contributions from the background. Instrumentally, this can be achieved through the ongoing development of the grazing incidence geometry PDF [151, 169]. On the other hand, coupling the PDF technique with other complementary tools (*e.g.*, XAS) can also facilitate the precise extraction of heterogeneities in MOFs [170].

The ongoing development in the aforementioned directions and beyond will create opportunities to further advance the fundamental understanding of MOF structures and properties, which will aid in identifying the active sites and rate-determining steps, subsequently contribute to the judicious design of MOF-based materials, ultimately promote the development of electrochemical devices involving MOFs and MOF-based composites for water applications. With the collective efforts within the research and industrial communities, it is anticipated that MOFs will play a more critical role in addressing the challenges around the energy–water systems as well as developing a circular economy.
